# Feasibility and impact of a large language model pipeline for surgical trial abstracts

**DOI:** 10.1038/s41746-026-02788-y

**Published:** 2026-05-26

**Authors:** Obafunsho John Abiola, Dmitri Nepogodiev, James Glasbey, Omar Omar, Cortland Linder, Virginia Ledda, Aneel Bhangu

**Affiliations:** Surgical Data Institute, University of Birmingham, Birmingham, UK

**Keywords:** Computational biology and bioinformatics, Health care, Mathematics and computing, Medical research

## Abstract

Abstracts of surgical randomised controlled trials (RCTs) are widely read but often omit key methodological details despite CONSORT guidance. We evaluated whether a tightly constrained large language model (LLM) pipeline could improve completeness and readability at scale. We conducted a three-phase in silico study of consecutive surgical RCTs indexed in PubMed (2005–2025) with open-access full texts in PubMed Central. A 14-item CONSORT-derived rubric (maximum 25 points) was developed and validated against expert scoring, demonstrating good agreement (concordance correlation coefficient 0.71, 95% CI 0.44–0.86) and high reproducibility (intraclass correlation coefficient 0.91, 95% CI 0.80–0.96). An automated pipeline using GPT-4o (OpenAI) generated rewritten abstracts from full texts under strict non-fabrication constraints. Among 651 RCTs, original abstracts showed low completeness (mean 9.06/25, 95% CI 8.58–9.53). Rewriting significantly improved completeness (mean increase 7.40 for 250-word and 8.06 for 300-word versions; both *p* < 0.0001), with gains across all CONSORT domains, particularly randomisation, harms, and trial registration. Readability improved slightly and correlated with completeness. A constrained LLM pipeline can substantially enhance the completeness of surgical RCT abstracts at scale, with potential applications in authoring, peer review, and editorial workflows, provided appropriate human oversight.

## Introduction

Transparent and complete reporting of randomised controlled trials (RCTs) is essential for trustworthy evidence synthesis and clinical decision-making. The CONSORT checklist specifies the minimum information that trial reports and abstracts should contain, including 17 items across the domains of design, randomisation, blinding, numbers randomised and analysed, harms, registration, and funding^1^. However, adherence remains inconsistent. A large-scale evaluation of more than 21,000 RCTs found that key CONSORT items remain frequently under-reported^2^.

Within surgery, reporting is weaker: of 356 surgical RCT abstracts reviewed before and after CONSORT, mean adherence was only 6.1/15 and 8.1/15 items, with participants, randomisation, blinding, analysis numbers, trial registration, and funding reported in less than half of the abstracts, even later^3^. This is especially concerning in global surgery, where many rely on abstracts because full texts are inaccessible, most often due to paywalls.

Large language models (LLMs) have been proposed as tools to support scientific writing and editing, but current evidence suggests that naïve use does not reliably improve the quality of trial abstracts. In one study, RCT abstracts generated de novo by GPT (OpenAI) from full trial reports were judged more readable than the originals, yet had substantially lower CONSORT adherence^4^. Another experiment showed that human reviewers frequently misclassified LLM-generated abstracts as real, despite the presence of fabricated numerical results, highlighting the risk of undetected hallucinations^5^. A further study asked GPT-4 to “improve” researchers’ own conference abstracts; the AI-edited versions were rated worse than the originals, although the final abstracts that authors produced after selectively editing the AI output were rated highest, suggesting that LLMs may be useful as assistants rather than autonomous writers^6^. Parallel work has used LLM-based pipelines to audit reporting quality at the article level across thousands of RCTs, confirming widespread under-reporting of core CONSORT items but not attempting to rewrite abstracts to address these gaps^2^. Overall, existing studies show that LLMs can generate plausible and sometimes more readable text, but there is limited evidence that they can systematically improve reporting completeness in real journal abstracts.

In this study, we use surgical RCT abstracts as an exemplar to test whether a carefully designed LLM-based approach can enhance reporting at scale. Surgical trials encompass heterogeneous procedures, complex multidisciplinary care pathways, and diverse trial designs, making their abstracts a challenging and clinically important benchmark for reporting quality^3^. We aimed to automate both the assessment and rewriting of published surgical RCT abstracts using GPT-4o (OpenAI), and to do so across a large corpus retrieved from PubMed, to test whether improvements can be efficiently created across multiple article types. Specifically, we sought to (1) develop a graded CONSORT-derived rubric for abstract completeness; (2) validate LLM-based scoring against experts; and (3) apply a structured prompting strategy to generate revised abstracts, comparing reporting completeness and readability between original and rewritten versions.

## Results

### Development and validation

In Phase 1, the CONSORT-derived rubric and scoring prompts underwent iterative refinement using five purposefully selected abstracts. Item-level discrepancies between reviewers, as well as between human and initial LLM scoring, guided the clarification of scoring anchors and evidentiary thresholds before formal validation.

**Fig. 1:**
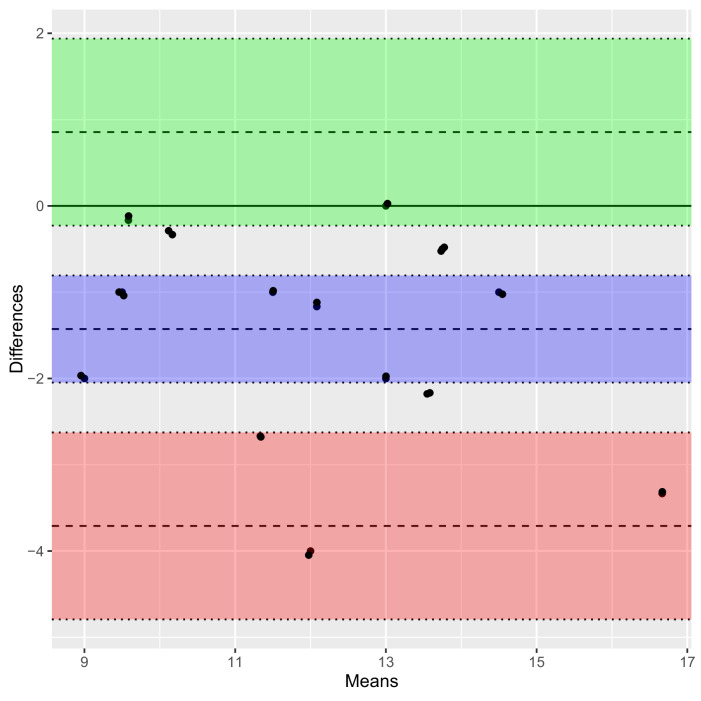
Bland–Altman plot comparing GPT-4o and human scoring for CONSORT completeness. Each point represents one abstract, plotted as the difference between mean GPT-4o and mean human scores (y-axis) against the mean of the two methods (x-axis); the mean bias was small, with limits of agreement indicating acceptable spread across the scoring range and no evidence of systematic directional bias, consistent with the concordance correlation coefficient of 0.71 (95% CI 0.44–0.86) reported between methods.

During the validation phase (*n* = 20 abstracts), inter-rater reliability between the two expert reviewers was high (ICC 0.937, 95% CI 0.835–0.977; two-way mixed-effects model, single-measure, absolute agreement). The reproducibility of LLM scoring across three independent runs was similarly high (ICC 0.907, 95% CI 0.802–0.963; two-way mixed-effects model, single-measure, absolute agreement). The agreement between mean human and mean model scores produced a concordance correlation coefficient (CCC) of 0.71 (95% CI 0.44–0.86), reflecting moderate-to-good concordance. Bland–Altman analysis showed a small mean bias between model and human scores, with limits of agreement consistent with the observed CCC and no evidence of systematic bias across the scoring range (Fig. 1).

### Study sample

Of the 1066 surgical RCTs identified, 651 (61%) met the open-access full-text requirement and were included in the analysis. These 651 trials had a corresponding PMC record with usable full-text XML, and are the basis for the rewriting analysis.

### Baseline reporting completeness

**Fig. 2:**
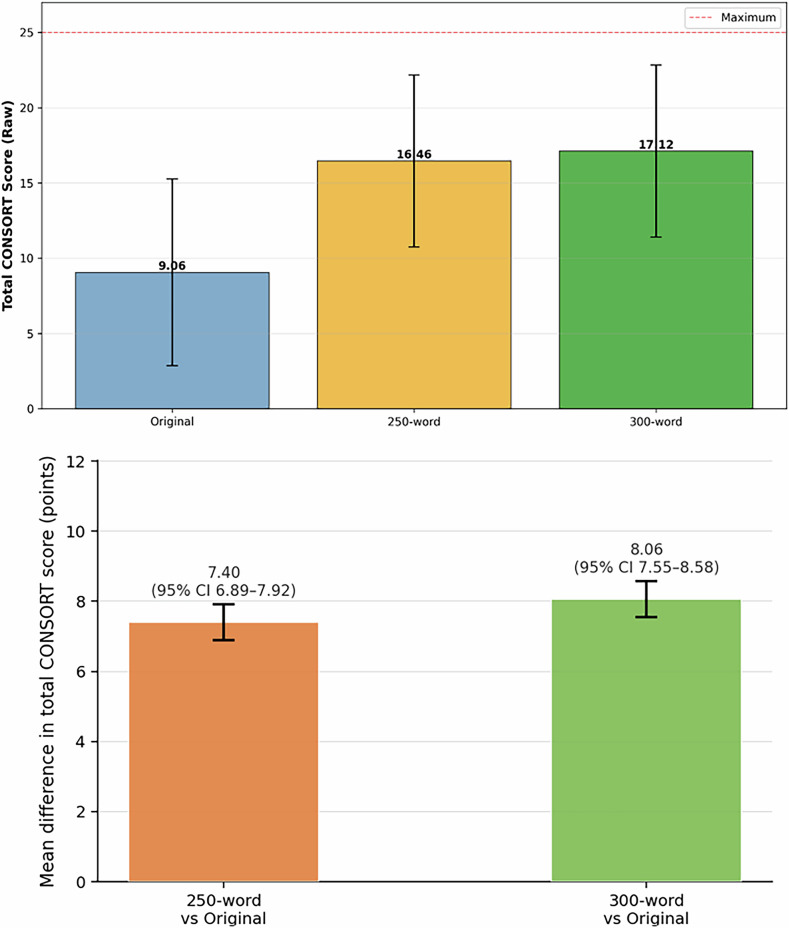
Overall CONSORT completeness scores across abstract versions. **A** Mean total CONSORT scores (maximum = 25) for original, 250-word, and 300-word rewritten abstracts, demonstrating significantly higher completeness in both rewritten formats; **B** mean paired differences in total CONSORT score (±95% CI) between rewritten and original abstracts, with improvements of ~+7 to +8 points for both formats.

The original abstracts of surgical randomised controlled trials exhibited significant under-reporting. The mean CONSORT completeness score was 9.06 points (SD 6.21, 95% CI 8.58–9.53), reflecting consistently low baseline reporting quality within the cohort. Both rewritten formats approached, but did not reach, the maximum possible score of 25. The mean CONSORT completeness for the 250-word rewritten version was 16.46 points (SD 5.72; 95% confidence interval: 16.02–16.90), while the 300-word rewritten version achieved a mean of 17.12 points (SD 5.72; 95% confidence interval: 16.68–17.56) (Table 1 and Fig. 2A).

**Table 1:** Resulting completeness levels

Version	Mean	SD	95% CI
Original	9.06	6.21	8.58–9.53
250-word	16.46	5.72	16.02–16.90
300-word	17.12	5.72	16.68–17.56

### Impact of LLM rewriting on reporting completeness

Both rewritten versions demonstrated substantial and statistically significant improvements in reporting completeness compared to the original abstracts. The 250-word abstracts increased total CONSORT scores by a mean of +7.40 points (95% CI 6.89–7.92), indicating a large effect size (Cohen’s *d* = 1.11). This improvement was confirmed by a paired *t*-test (*t*(650) = 28.20, *p* < 0·0001). The 300-word versions achieved an even greater gain of +8.06 points (95% CI 7.55–8.58), also reflecting a large effect size (Cohen’s *d* = 1.20) and high statistical significance (*t*(650) = 30.59, *p* < 0·0001) (Table 2 and Fig. 2B).

**Table 2:** Paired comparisons of completeness improvements

Comparison	*n*	Mean difference	95% CI	*t*-statistic	*p* value	Cohen’s *d*
250-word vs original	651	7.40	6.89–7.92	28.20	6.7 × 10⁻¹¹⁵	1.11
300-word vs original	651	8.06	7.55–8.58	30.59	5.3 × 10⁻¹²⁸	1.20

### Item-level effects of LLM rewriting

**Fig. 3:**
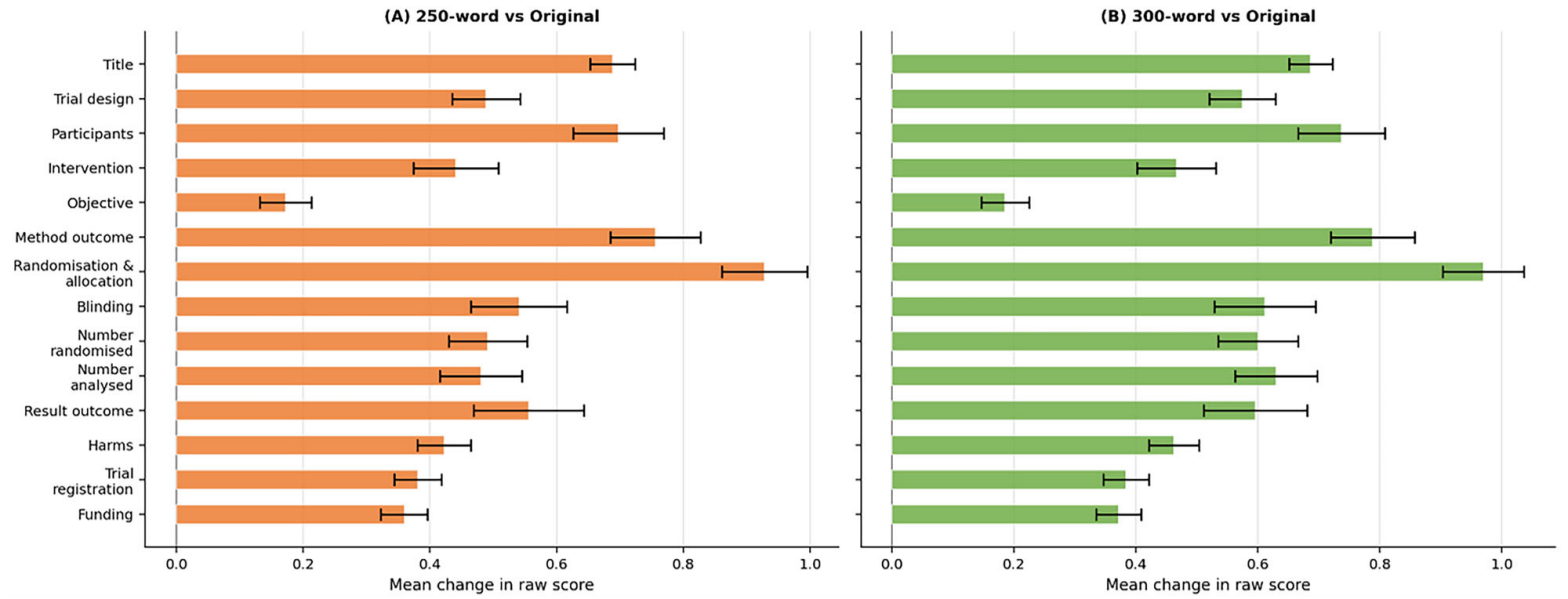
Item-level changes in raw CONSORT scores following LLM rewriting. Mean change in raw item scores for **A** 250-word and **B** 300-word rewritten abstracts compared with originals, showing consistent gains across all 14 CONSORT items, with the largest improvements in randomisation and allocation concealment, methods and primary outcome, and participants.

**Fig. 4:**
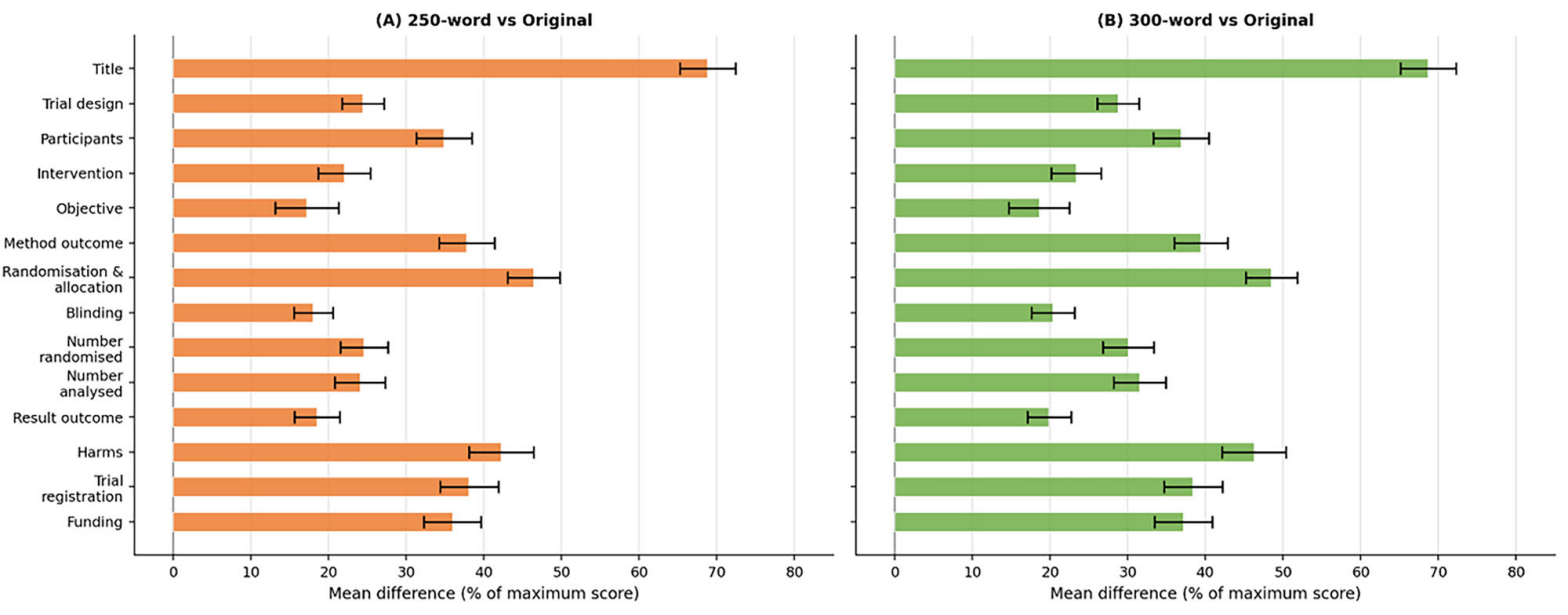
Item-level improvements expressed as a percentage of the maximum possible score. Mean differences (±95% CI) in percentage-completeness per CONSORT item for **A** 250-word and **B** 300-word rewritten abstracts relative to originals, with the greatest gains observed for title identification, randomisation and allocation, and harms items.

Substantial improvements were observed across all 14 CONSORT items. The most significant gains occurred in Randomisation and Allocation concealment (250-word: +0.93; 300-word: +0.97), Methods and Primary outcome reporting (250-word: +0.76; 300-word: +0.79), as well as in Participants, Title identification, and numerical items such as numbers randomised, and numbers analysed (Figs. 3 and 4).

**Fig. 5:**
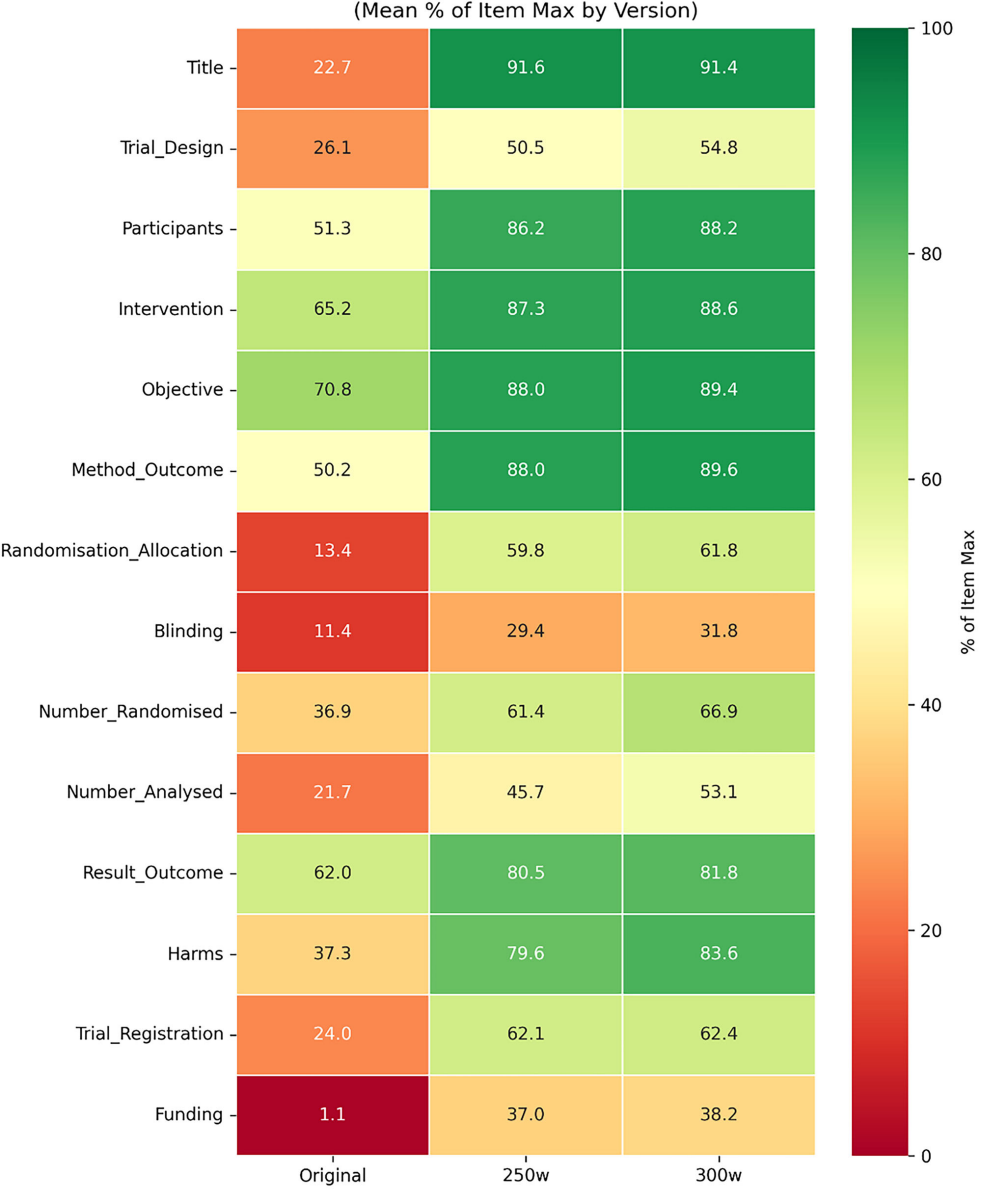
Heatmap of average percentage-completeness per CONSORT item across abstract versions. Colour intensity reflects the mean percentage of maximum score per item for original, 250-word, and 300-word abstract versions, illustrating broad and consistent improvements across all domains, with the largest gains in title identification, randomisation and allocation, and methods and primary outcome reporting.

When assessed using percentage-completeness values, the largest increases were observed in Randomisation and Allocation, which rose by 46.4 percentage points in the 250-word versions and 48.5 percentage points in the 300-word versions. Marked improvements were also evident in Method and Primary Outcome reporting (37.8 to 39.4 percentage points), Participants (34.9 to 36.9), Title identification (68.7 to 68.8), and numerical items such as numbers randomised (24.6 to 30.0), and numbers analysed (24.0 to 31.5). Smaller but consistent improvements were noted in Objective (17.2 to 18.6) and funding (35.9 to 37.2), which are items more frequently reported in the original abstracts. Gains were marginally greater in the 300-word format, consistent with the increased word limit (Table 3a, b and Fig. 5).

**Table 3:** **a** Item-level Improvements in Reporting Completeness: 250-word rewrites vs original abstracts (*n* = 651). **b** Item-level improvements in reporting completeness: 300-word rewrites vs original abstracts (*n* = 651)

CONSORT item	Original mean (%)	250-word mean (%)	Δ 250-word (% points)	Cohen’s *d* (250)	95% CI	Effect	*p* (raw)	*p* (BH-adj)
a								
Title	22.7	91.6	+68.8	1.484	1.39 to 1.60	Large	<0.001	<0.001
Trial design	26.1	50.5	+24.4	0.700	0.60 to 0.77	Medium	<0.001	<0.001
Participants	51.3	86.2	+34.9	0.750	0.66 to 0.83	Medium	<0.001	<0.001
Intervention	65.2	87.3	+22.0	0.505	0.41 to 0.57	Medium	<0.001	<0.001
Objective	70.8	88.0	+17.2	0.323	0.22 to 0.38	Small	<0.001	<0.001
Method outcome	50.2	88.0	+37.8	0.819	0.72 to 0.90	Large	<0.001	<0.001
Randomisation & allocation	13.4	59.8	+46.4	1.056	0.96 to 1.15	Large	<0.001	<0.001
Blinding	11.4	29.4	+18.0	0.548	0.45 to 0.62	Medium	<0.001	<0.001
Number randomised	36.9	61.4	+24.6	0.615	0.52 to 0.69	Medium	<0.001	<0.001
Number analysed	21.7	45.7	+24.0	0.571	0.47 to 0.64	Medium	<0.001	<0.001
Result outcome	62.0	80.5	+18.5	0.492	0.40 to 0.56	Small	<0.001	<0.001
Harms	37.3	79.6	+42.2	0.776	0.68 to 0.85	Medium	<0.001	<0.001
Trial registration	24.0	62.1	+38.1	0.779	0.68 to 0.86	Medium	<0.001	<0.001
Funding	1.1	37.0	+35.9	0.749	0.65 to 0.82	Medium	<0.001	<0.001

CONSORT item	Original mean (%)	300-word mean (%)	Δ 300-word (% points)	Cohen’s *d* (300)	95% CI	Effect	*p* (raw)	*p* (BH-adj)
b								
Title	22.7	91.4	+68.7	1.479	1.38 to 1.60	Large	<0.001	<0.001
Trial design	26.1	54.8	+28.7	0.815	0.72 to 0.90	Large	<0.001	<0.001
Participants	51.3	88.2	+36.9	0.797	0.70 to 0.88	Medium	<0.001	<0.001
Intervention	65.2	88.6	+23.3	0.558	0.46 to 0.63	Medium	<0.001	<0.001
Objective	70.8	89.4	+18.6	0.365	0.27 to 0.42	Small	<0.001	<0.001
Method outcome	50.2	89.6	+39.4	0.884	0.79 to 0.97	Large	<0.001	<0.001
Randomisation & allocation	13.4	61.8	+48.5	1.126	1.03 to 1.22	Large	<0.001	<0.001
Blinding	11.4	31.8	+20.4	0.567	0.47 to 0.64	Medium	<0.001	<0.001
Number randomised	36.9	66.9	+30.0	0.705	0.61 to 0.78	Medium	<0.001	<0.001
Number analysed	21.7	53.1	+31.5	0.721	0.63 to 0.80	Medium	<0.001	<0.001
Result outcome	62.0	81.8	+19.9	0.542	0.45 to 0.61	Medium	<0.001	<0.001
Harms	37.3	83.6	+46.2	0.865	0.77 to 0.95	Large	<0.001	<0.001
Trial registration	24.0	62.4	+38.4	0.789	0.69 to 0.87	Medium	<0.001	<0.001
Funding	1.1	38.2	+37.2	0.769	0.67 to 0.85	Medium	<0.001	<0.001

*CI* 95% confidence interval (bootstrapped, 5000 iterations), *BH-adj* Benjamini–Hochberg false discovery rate adjusted *p* value.

Effect size thresholds (Cohen’s *d*): Small ≥0.20, Medium ≥0.50, Large ≥0.80. All BH-adjusted *p* values <0.001; no item lost significance after FDR correction across 14 comparisons.

After adjustment for multiple comparisons using the Benjamini–Hochberg procedure, all 14 CONSORT items remained statistically significant for both the 250-word and 300-word rewrites (all adjusted *p* < 0.001).

Effect sizes ranged from small to large. In the 250-word rewrites, large effects were observed for title (*d* = 1.48, 95% CI 1.39–1.60), randomisation and allocation (*d* = 1.06, 95% CI 0.96–1.15), and method outcome (*d* = 0.82, 95% CI 0.72–0.90). Most remaining items demonstrated medium effects (*d* = 0.49–0.78), while the objective (*d* = 0.32) showed a small effect. Effect sizes were consistently slightly larger in the 300-word rewrites across most items (Table 3a, b and Fig. 5).

**Fig. 6:**
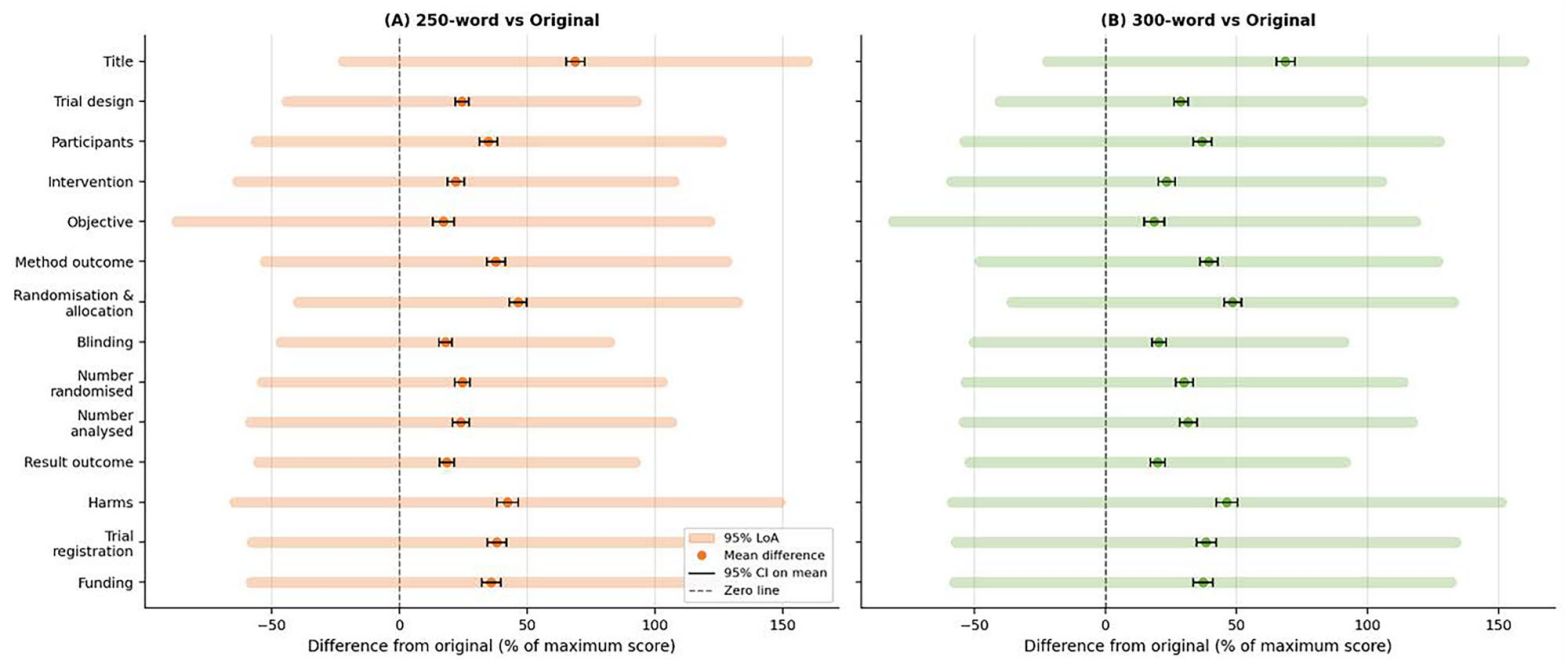
Item-level Bland–Altman analysis of CONSORT score changes following LLM rewriting, expressed as percentage of maximum score. Forest-plot-style Bland–Altman plots display, for each of the 14 CONSORT items, the mean difference (orange/green dot) and 95% CI on the mean (error bars) between rewritten and original abstract scores, alongside 95% limits of agreement (shaded band), for **A** 250-word and **B** 300-word rewrites; all mean differences were positive, confirming consistent improvement across every item, with the widest limits of agreement observed for Title identification and Harms, reflecting greater heterogeneity in baseline reporting of these items; the lower limit of agreement crosses zero for several items, indicating that a minority of individual abstracts scored lower after rewriting, though no systematic negative bias was observed at the group level.

Item-level Bland–Altman analyses are presented in Fig. 6. Across all 14 CONSORT items, rewritten abstracts demonstrated positive mean differences compared with the originals. The limits of agreement indicate variability in the magnitude of improvement across abstracts, with wider dispersion observed for items exhibiting larger absolute gains, such as Title identification and Randomisation and Allocation. Although the lower limits of agreement crossed zero for several items, indicating that a minority of individual abstracts scored lower after rewriting, no evidence of systematic negative bias was observed at the group level. The observed dispersion likely reflects heterogeneity in baseline reporting completeness, with greater variability in items that were infrequently reported in the original abstracts.

### Readability and length

Rewriting using large language models resulted in significant improvements in readability while maintaining scientific content. For the Flesch–Kincaid Grade Level, both rewritten versions produced easier to read text. The 250-word rewrites demonstrated a mean reduction of 1.06 grade levels (95% CI: –1.31 to –0.82), *t*(650) = −8.42, *p* < 0.0001, *d* = −0.33. The 300-word rewrites showed a mean reduction of 0.87 grade levels (95% CI: −1.11 to −0.62), *t*(650) = −6.96, *p* < 0.0001, *d* = −0.27. Improvements were slightly greater in the 250-word versions, indicating more aggressive syntactic simplification.

For Flesch reading ease, scores increased consistently: 250-word rewrites improved by 2.79 points (95% CI 1.65–3.93), *t* = 4.79, *p* < 0.0001, *d* = 0.19; 300-word rewrites improved by 2.24 points (95% CI 1.13–3.35), *t* = 3.96, *p* < 0.0001, *d* = 0.16. Although these improvements were modest in magnitude, they were systematic across the 651 trials.

**Fig. 7:**
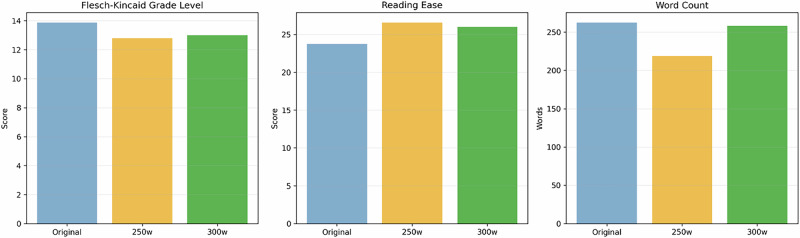
Readability characteristics across original and rewritten abstract versions. Bar plots show mean Flesch–Kincaid Grade Level, Flesch Reading Ease, and Word Count for original, 250-word, and 300-word versions; both rewritten formats demonstrated lower grade levels, higher reading ease scores, and reduced or preserved word counts relative to the originals.

**Fig. 8:**
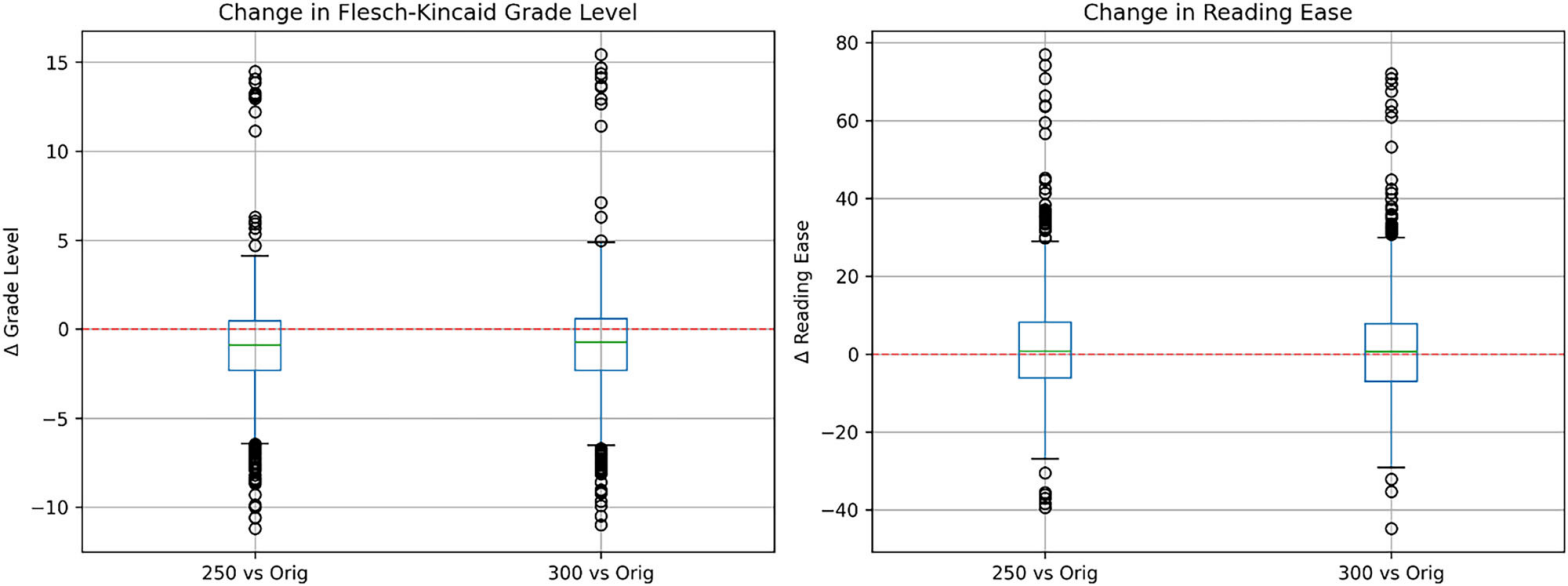
Distribution of paired changes in readability metrics following LLM rewriting for 250-word and 300-word abstract versions relative to originals. Box plots display paired changes (Δ) in Flesch–Kincaid Grade Level (left) and Flesch Reading Ease (right) for 250-word and 300-word rewrites relative to originals; the red dashed line indicates zero change, and the green line indicates the median; median grade level reductions were modest but consistently negative across both formats, indicating that most abstracts became easier to read, while median reading ease improvements were near zero, with statistically significant mean improvements driven by a subset of abstracts showing large positive gains; substantial variability and outliers in both directions reflect heterogeneity in source abstract complexity.

**Fig. 9:**
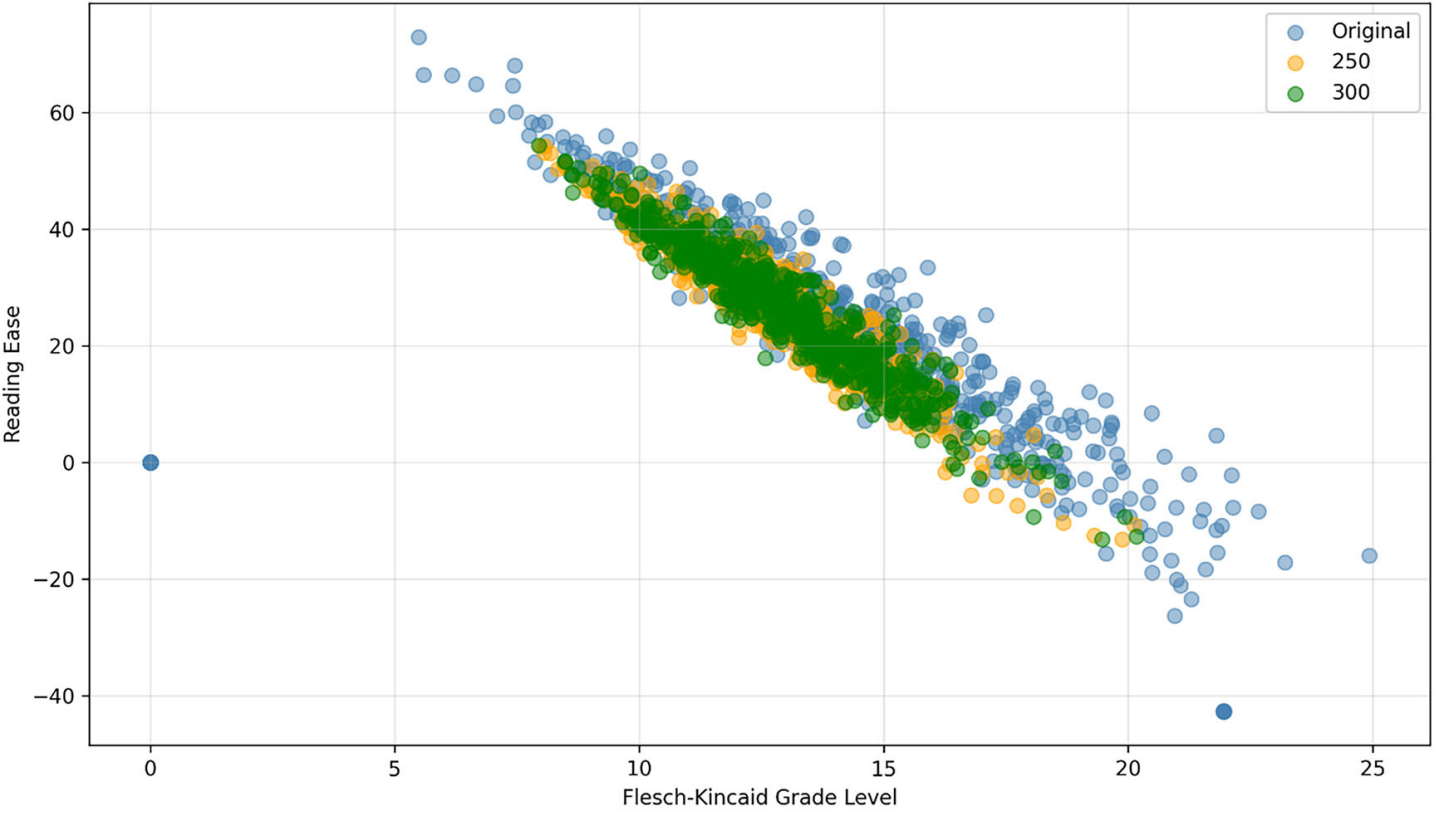
Correlation between Flesch–Kincaid grade level and Flesch reading ease across abstract versions. Each point represents an individual abstract, coloured by version (original, 250-word, or 300-word), illustrating the expected inverse relationship between grade level and reading ease and confirming that LLM rewriting shifted abstracts towards lower grade levels and higher reading ease scores.

Regarding word count, the 250-word rewrites showed the greatest reduction: mean change −43.33 words (95% CI −51.82 to −34.85), *t* = −10.03, *p* < 0.0001, d = −0.39. The 300-word rewrites exhibited a mean change of −4.17 words (95% CI −12.53 to +4.18), *t* = −0.98, *p* = 0.33 (not significant), d = −0.04 (Tables 4–6 and Figs. 7–9). Therefore, the 300-word format preserved the original length while maintaining or slightly improving readability.

**Table 4:** Readability and structural characteristics of original and rewritten abstracts

Version	Mean Flesch–Kincaid grade level	Mean Flesch reading ease	Mean word count	SD (word count)	Median word count
Original	13.86	23.76	262.3	108.9	258
250-word	12.80	26.55	218.9	24.0	221
300-word	12.99	26.00	258.1	23.7	258

**Table 5:** Comparison of readability (Flesch–Kincaid grade level & Flesch reading ease) between original and rewritten abstracts

Comparison	Metric	Mean Δ FKGL	95% CI	*t*-statistic	*p* value	Cohen’s *d*
250 vs original	FKGL	−1.06	[−1.31, −0.82]	−8.42	2.4 × 10^−^^16^	−0.33
300 vs original	FKGL	−0.87	[−1.11, −0.62]	−6.96	8.3 × 10^−^^12^	−0.27
250 vs original	FRE	+2.79	[1.65, 3.93]	+4.79	2.1 × 10^−^^6^	+0.19
300 vs original	FRE	+2.24	[1.13, 3.35]	+3.96	8.2 × 10^−^^5^	+0.16

**Table 6:** Summary of comparative readability and structural metrics across abstract versions

Metric	Original	250-word	300-word	Δ vs Original	*p* value	Effect (d)	Interpretation
FKGL	13.86	12.80	12.99	−1.06 to −0.87	<0.001	0.27–0.33	Easier syntax
Reading Ease	23.76	26.55	26.00	+2.24 to +2.79	<0.001	0.16–0.19	Slightly more readable
Word Count	262.3	218.9	258.1	−43.3 (250-word), −4.2 (300-word)	<10⁻²¹ (250); n.s. for 300	0.39 (250)	Significant compression at 250 words

### Correlation between reporting completeness, readability, and abstract length

The relationships among CONSORT reporting completeness, readability metrics, and abstract length were analysed to assess whether clearer or longer abstracts are inherently associated with higher completeness scores. Correlations were calculated across all three versions of each abstract (original, 250-word, and 300-word; 1953 observations).

Both readability and length demonstrated measurable associations with CONSORT completeness. Flesch–Kincaid grade level exhibited a moderate negative correlation with completeness (*r* = –0.36, 95% CI –0.40 to –0.32, *p* < 0.0001), indicating that abstracts written at a lower grade level tended to have more complete reporting. Flesch Reading Ease showed a moderate positive correlation (*r* = 0.38, 95% CI 0.34 to 0.42, *p* < 0.0001), suggesting that clearer and more readable writing was associated with better adherence to CONSORT criteria.

**Fig. 10:**
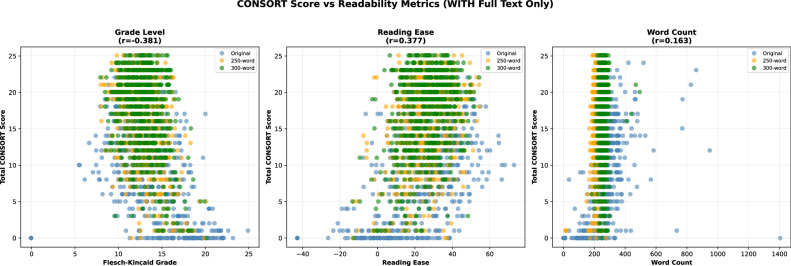
Associations between CONSORT completeness and readability metrics across all abstract versions. Multipaneled scatter plots show relationships between total CONSORT score and **A** Flesch–Kincaid grade level, **B** Flesch reading ease, and **C** Word count across 1953 observations (original, 250-word, and 300-word versions); readability indices showed moderate associations with completeness, while word count demonstrated a modest positive correlation (*r* = 0.29).

Word count demonstrated a modest positive correlation (*r* = 0.29, 95% CI 0.25 to 0.33, *p* < 0.0001), indicating that longer abstracts tended to report more CONSORT items, although length alone accounted for only a small proportion of the variability in completeness (Table 7 and Fig. 10). The 300-word rewritten abstracts improved completeness without substantially exceeding the original abstract length. Overall, readability, rather than verbosity, demonstrated a stronger relationship with reporting completeness.

**Table 7:** Correlation between readability metrics and CONSORT completeness scores

Metric	Original Pearson correlation (*r*)	250-word Pearson correlation (*r*)	300-word Pearson correlation (*r*)	Pooled approx (mean *r*)	Direction	Interpretation
Flesch–Kincaid grade level	−0.386	−0.356	−0.332	~ −0.36	Moderate negative	More complex syntax weakly associated with lower completeness.
Reading ease	+0.508	+0.312	+0.302	~ +0.37	Moderate positive	Abstracts that are easier to read tend to have higher reporting completeness.
Word count	+0.417	+0.328	+0.119	~ +0.29	Moderate positive	Longer abstracts show higher completeness, suggesting compression restricts reporting.

In summary, clarity (lower grade level, higher reading ease) is more strongly associated with better CONSORT reporting than length, and length alone provides minimal predictive value for completeness. Model rewriting enhances completeness while maintaining or slightly improving readability, primarily through structural reorganisation, explicit phrasing, and improved coherence, rather than through increased verbosity.

## Discussion

This study shows that a large language model, when tightly constrained and supplied with underlying trial text, can systematically improve the reporting completeness of surgical randomised controlled trial (RCT) abstracts and do so in an automated way. Using a rigorously validated CONSORT-derived rubric and an auditable, large-scale scoring and rewriting workflow, GPT-4o produced consistent gains across all 14 reporting items, particularly those known to be frequently under-reported in surgical trials, including randomisation, allocation concealment, harms, and trial registration.

Our automated pipeline efficiently demonstrated that improvements were observed across a large and methodologically diverse set of surgical trials spanning two decades, supporting robustness across subspecialties and trial designs, this pipeline can be used at scale when needed. These results provide evidence that a tightly constrained, source-bound LLM workflow can enhance real-world reporting quality at scale, addressing substantive reporting deficits rather than merely improving style.

Our work extends and contrasts with the emerging literature on AI-assisted scientific writing. Speich and colleagues showed that surgical abstracts frequently omit essential CONSORT items even after publication of the checklist, with fewer than half reporting blinding, numbers analysed, or trial registration^3^. Hopewell et al. similarly documented widespread deficits in RCT abstract reporting across medical fields^1^.

Previous evaluations of LLM-generated abstracts have not demonstrated improvements in reporting quality. Studies in which GPT-generated RCT abstracts were produced from full trial reports found that these outputs were often more readable but substantially less compliant with CONSORT reporting standards^4^. Reviewers have also misclassified GPT-generated abstracts as authentic despite fabricated numerical results^5^. Experiments using GPT-4 for abstract revision reported that AI-edited versions did not outperform clinician-written originals, although authors sometimes found the suggestions useful^6^.

In parallel, large-scale assessments have shown that LLM-based auditing pipelines can detect under-reporting across tens of thousands of RCTs, highlighting the potential for automation in quality evaluation^2^. However, these approaches focused on auditing rather than rewriting abstracts to address reporting gaps. The present study therefore contributes novel evidence that LLMs can be used not only to evaluate reporting completeness but also to generate structured revisions that substantially improve reporting quality.

Although we used a rigorous multistage protocol, LLMs remain intrinsically prone to hallucinations, producing confident yet incorrect statements. This issue cannot be fully eliminated even with deterministic sampling and evidence citation, and recent analyses argue that hallucination is an unavoidable property of probabilistic text generation models^7^. Empirical studies in biomedical contexts similarly document persistent fabrication of references, numbers, and mechanistic details despite carefully written prompts^8^. Our pipeline reduces but cannot abolish this risk, and subtle inaccuracies may have gone undetected.

The rubric measured reporting completeness rather than factual correctness, meaning that a highly scored abstract could still misrepresent a trial’s findings. Although the rewriting pipeline was strictly source-grounded and manually inspected during development, we did not conduct a large-scale numerical audit of all rewritten abstracts. High completeness scores, therefore, do not guarantee the correctness of reported values, and human oversight remains essential. This distinction has been increasingly emphasised in recent commentary on AI-assisted scientific writing^9^.

The study sample, although large for an abstract-level intervention, was limited to surgical RCTs and English-language abstracts indexed in PubMed Central with open-access full text. Restriction to PMC-indexed open-access full texts may introduce systematic selection bias, potentially over-representing journals with open-access policies, English-language publications, or trials from higher-resource settings. As eligibility was determined by machine-readable full-text availability required for the intervention, the sample may not fully represent subscription-based or multilingual RCT literature. Generalisability to subscription-only journals, other specialties, languages, and non-randomised designs remains uncertain. Future validation across broader databases and alternative access pathways is warranted to assess external validity.

The rubric represents a pragmatic operationalisation of CONSORT reporting principles rather than a formally developed psychometric scale. It was designed to enable structured and reproducible assessment of abstract completeness at scale. Although reliability was formally evaluated, we did not undertake full psychometric validation (e.g., factor analysis or construct modelling), as the underlying conceptual framework is defined by CONSORT. Although expert inter-rater agreement was excellent, expert scoring remains a surrogate reference rather than an absolute ground truth. Importantly, reporting completeness should not be conflated with methodological quality or risk of bias; appraisal frameworks such as JBI or Cochrane tools evaluate internal validity, whereas our rubric assesses transparency of reporting. Accordingly, this study does not evaluate trial quality or risk of bias, and improvements in reporting completeness should not be interpreted as improvements in study design, methodological rigour, or validity. This limits the ability to infer whether rewritten abstracts better reflect high-quality trials.

Finally, only a single LLM configuration (GPT-4o) was evaluated. Model performance may vary across architectures or future model updates, and the results should therefore be interpreted as applying to the specific model configuration tested in this study.

Automation bias is another concern. Polished AI-generated text may engender over-trust, creating a risk of insufficient scrutiny during peer review or editorial assessment^10^. Current guidance from the International Committee of Medical Journal Editors (ICMJE) and the Committee on Publication Ethics (COPE) emphasises that AI tools should not be listed as authors and that human authors retain full responsibility for the accuracy, integrity, and attribution of all submitted work. In this study, the LLM functioned solely as an assistive tool within a constrained and auditable workflow; it did not meet authorship criteria, and ultimate responsibility for all rewritten content rests with the named human authors. Any deployment of such systems in editorial or author workflows should require mandatory human verification prior to submission or publication.

Regarding copyright, this study used publicly accessible abstracts and PubMed Central full texts for in silico transformation without redistribution of copyrighted material. The rewritten abstracts were generated for methodological evaluation rather than republication. Nevertheless, journal policies regarding AI-assisted writing vary, and implementation of such tools should comply with publisher-specific disclosure and attribution requirements.

Broader systemic issues include the possibility that AI-assisted writing may accelerate publication pressure or exacerbate inequities between researchers with and without access to advanced tools. Finally, although our large-scale application demonstrates technical feasibility, this study did not evaluate downstream effects in editorial workflows, clinical decision-making, or evidence synthesis.

LLM-assisted tools could support authors by identifying missing CONSORT items and offering structured rewrites that preserve scientific meaning while enhancing transparency. Editorial and journal workflows that incorporate LLM tools are likely to achieve the greatest improvements when models are provided with full-text submissions, enabling more complete reporting in abstracts without requiring additional manual rewriting. Because the model was provided with underlying trial text, aggregated improvements may underestimate the potential gains achievable when full manuscripts are available at submission. Editors and peer reviewers could use such systems as adjuncts to manual assessment, helping standardise abstract reporting and reduce avoidable omissions that impair evidence interpretation.

Because the workflow is fully automated and has low marginal computational cost (approximately $0.12 per abstract), such pipelines could be implemented at scale within editorial or research workflows. Journals and professional societies could use it to monitor reporting quality across portfolios or specialties, repeating analyses as new manuscripts accrue without additional manual scoring. In global surgery, where clinicians often rely on abstracts due to restricted access to full texts, improving reporting completeness could reduce disparities in access to reliable trial information.

Automated scoring pipelines could also enable reporting guideline groups to track adherence trends longitudinally and provide evidence-based feedback to authors, reviewers, and editors.

Future work should evaluate the generalisability of this approach in other clinical specialties, languages, and study designs, including pragmatic and cluster trials. Because LLM performance may vary across model generations, future benchmarking studies should evaluate the robustness of scoring and rewriting performance across alternative architectures and subsequent model updates.

Additional work should be done to evaluate independent scoring models or human-only scoring to eliminate any residual circularity and confirm the generalisability of scoring behaviour. Randomised evaluations embedded within editorial workflows could test AI-assisted authoring, peer review, and editorial triage to determine real-world benefits and risks.

Although completeness scores improved substantially, they do not guarantee that reported methods or results are correct; they only indicate that required reporting elements are present. Future research should therefore formally quantify numerical error rates in larger samples prior to deployment of such systems in editorial workflows. We did not undertake full psychometric validation even though reliability of the rubric was formally evaluated. Future work could explore additional validation approaches in other specialties or reporting contexts.

Finally, similar constrained LLM workflows could be evaluated for improving completeness and clarity beyond abstracts, including full trial reports and CONSORT, PRISMA, or SPIRIT-governed sections^11^. As LLMs continue to evolve, ensuring transparency, version control, traceable outputs, and human oversight will remain essential to their responsible deployment in scientific communication. Automated pipelines such as the one described here offer a practical way to retest and recalibrate performance as each new model generation appears.

## Methods

### Study design and overall approach

**Fig. 11:**
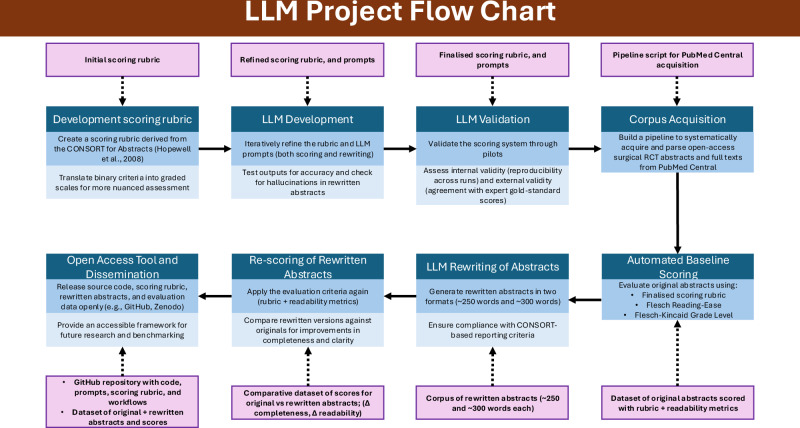
Overview of the LLM-based abstract rewriting and evaluation pipeline. The pipeline proceeds across four sequential stages (left to right): Development scoring rubric (iterative derivation of a graded 14-item CONSORT rubric); LLM development (refinement of scoring and rewriting prompts with hallucination checks); LLM validation (assessment of internal reproducibility and external agreement with expert gold-standard scores); and corpus acquisition (systematic retrieval of open-access surgical RCTs and full texts from PubMed Central). These stages feed into three parallel processing streams: automated baseline scoring (original abstracts scored using the finalised rubric and readability metrics), LLM rewriting of abstracts (generation of ~250-word and ~300-word rewrites constrained to CONSORT-based reporting criteria), and re-scoring of rewritten abstracts (rubric and readability re-evaluation), which together produce a comparative dataset of completeness and readability changes (Δ). Outputs are made openly available via GitHub and Zenodo to support reproducibility and future benchmarking.

We assessed whether a large language model (LLM) could enhance the reporting quality of surgical randomised controlled trial (RCT) abstracts using a retrospectively assembled open-access dataset. The protocol specified three sequential phases: development, validation, and large-scale application, each involving predefined steps for data handling, LLM prompting, scoring, and statistical analysis. Figure 11 outlines the computational workflow.

### Identification of open-access surgical RCTs

We identified consecutive surgical randomised controlled trials (RCTs) indexed in PubMed from 2005 to 2025 using a predefined database query provided in the Supplement (Supplementary Note 2). For each PubMed record, the PubMed Identifier (PMID), which uniquely identifies articles in PubMed, was mapped to a PubMed Central (PMC) identifier through the NCBI ELink service, a tool that connects records across databases from the National Center for Biotechnology Information (NCBI). Trials were considered open access if a PMC record existed and the full-text XML (machine-readable article format) could be retrieved and parsed. Full texts were obtained only from PMC (a free digital archive of biomedical literature). PubMed served solely as a citation index to identify eligible trials.

Articles without a PubMed Central (PMC) record, or those where XML (extensible markup language) file retrieval or parsing failed, were excluded from the rewriting analysis. To confirm that these exclusions indicated a real lack of open-access full text, we manually checked a random sample of excluded studies. We did this by accessing the PubMed record and confirming that there was no PMC link, indicating that the article was not in the free PubMed Central archive or a free publisher-provided PDF. Only clinical trials with full-text articles available in open access were included for scoring and rewriting.

### Objectives

The primary objective was to determine whether a carefully prompted LLM could enhance CONSORT reporting completeness and readability when rewriting surgical RCT abstracts from the underlying trial reports. Secondary objectives were to develop and validate a graded CONSORT-derived scoring rubric, to assess the reproducibility and agreement of LLM-based scoring relative to experts, and to quantify item-level changes in completeness and changes in readability metrics between original and rewritten abstracts.

### Protocol and reporting

A detailed protocol, including the rubric specification and prompting templates, is provided in the Supplement (see Supplementary Notes 2 and 9) with key study definitions and terminology used throughout the manuscript provided in Table 8. The study followed emerging guidance for AI prediction and evaluation studies, with emphasis on transparent description of data sources, model configuration, prompting, and evaluation procedures. The study was designed, conducted, and reported in accordance with the TRIPOD-LLM reporting guideline for studies involving large language models, and a completed TRIPOD-LLM checklist is provided in the Supplement (see Supplementary Note 1).

**Table 8:** Study definitions

Term	Definition
Scoring rubric completeness	Total score across 14 reporting items adapted from CONSORT, graded 0–1, 0–2, or 0–3 per domain (maximum = 25).
Readability	Measured using Flesch–Kincaid grade level and Flesch reading ease, supplemented by word count.
Rewriting formats	Each abstract was rewritten in two formats: ≈250 words and ≈300 words, both following a structured template.
BMRI structure	Background, Methods, Results, Interpretation—applied to all rewritten abstracts.
Development set	Five manually annotated abstracts scored by two surgical experts; used for iterative refinement of prompts and rubric.
Validation set	Twenty abstracts were manually annotated by experts and scored by GPT three times to test reproducibility and agreement with human scores.

### Phase 1: development of the rubric, prompts, and scoring workflow

In the development phase, we constructed a scoring rubric and a prompting strategy that could be applied consistently by both human raters and the LLM. Five English-language surgical RCT abstracts were purposefully sampled to capture a variety of abstract structures and reporting completeness. Eligibility required that the abstract describe a randomised trial of a surgical or perioperative intervention, be written in English, and have an accessible full-text PDF.

Two experts independently scored each abstract using a prototype 14-item rubric based on the CONSORT checklist, created by turning each checklist point into a specific scoring anchor. This rubric was designed as an operationalised, graded implementation of the established CONSORT for Abstracts guidance, rather than as a new reporting standard.

Each item was graded on a scale from 0 to 3, reflecting the explicitness, completeness, and precision of reporting. A score of 0 indicated absence of required information; (1) indicated vague, incomplete, or implied reporting; (2) denoted partial but substantively informative reporting; and (3) represented full adherence with explicit CONSORT-compliant detail. Item definitions and thresholds were drafted from the Hopewell et al. Explanation and Elaboration guidance and subsequently adapted for surgical RCT abstracts.

The prototype rubric underwent iterative refinement through consensus discussions following rater discrepancies, including wording adjustments, clarification of evidence requirements, and harmonisation of partial-credit rules. These modifications ensured the rubric captured clinically meaningful variation in reporting while remaining practical for consistent application. The rubric items covered: title identification as a randomised trial; trial design; participant eligibility and setting; intervention and comparator; objectives; primary outcome and timeframe; randomisation and allocation concealment; blinding; numbers randomised and analysed; results and effect estimates; harms; trial registration; and funding.

Following human scoring, GPT-4o (OpenAI; model version gpt-4o-2024-08-06) was then used to score the same five abstracts in three independent runs using an initial scoring prompt. For each item, the model was required to produce a numerical score and cite at least one verbatim excerpt from the abstract as supporting evidence.

Discrepancies between human and model scores, particularly for blinding, allocation concealment, and numbers analysed, were used diagnostically to refine scoring anchors and clarify evidentiary thresholds prior to formal validation, resulting in the revision of both the rubric wording and the prompt structure. We iteratively strengthened instructions to avoid speculation, clarified what constituted sufficient evidence for each score level, and enforced a strict JSON schema for outputs to facilitate automatic parsing and to prevent free-text commentary.

This process yielded a finalised 14-item CONSORT-derived rubric with a maximum score of 25, a scoring prompt designed to return structured scores with supporting quotations, and a rewriting prompt that enforced a Background–Methods–Results–Interpretation (BMRI) structure, explicit inclusion of CONSORT items when present in the source, and omission or neutral phrasing where information was unavailable.

No model fine-tuning or parameter optimisation was performed during this phase; modifications were limited to rubric definitions and prompt wording applied to a fixed base model configuration. Item-level human and GPT-4o scores for the five development-set abstracts are summarised in Table 9. The development sample size (*n* = 5 abstracts) was intentionally small and purposively selected. The aim of this phase was not statistical estimation but qualitative refinement of rubric definitions, scoring anchors, and prompt instructions prior to larger-scale evaluation. This approach reflects a pilot, iterative development stage used to identify sources of disagreement and stabilise scoring criteria prior to formal validation. Full rubric definitions and final prompt templates are provided in the Supplement (Supplementary Notes 3–6), and the mapping between rubric items and CONSORT checklist elements is provided in Supplementary Note 6.

**Table 9:** Development set item-wise scores for five surgical RCT abstracts

Item	Paper 1 (Human/GPTMean)	Paper 2	Paper 3	Paper 4	Paper 5
Title (max = 1)	1.0/1.0	1.0/1.0	1.0/1.0	1.0/ 1.0	1.0/1.0
Trial design (max = 2)	2.0/2.0	1.0/1.0	1.0/1.0	1.0/1.0	1.0/1.0
Participants (max = 2)	2.0/2.0	1.0/1.0	2.0/2.0	0.5/1.0	2.0/2.0
Intervention (max = 2)	2.0/2.0	2.0/2.0	2.0/2.0	2.0/2.0	2.0/2.0
Objective (max = 1)	1.0/1.0	1.0/1.0	1.0/1.0	1.0/1.0	1.0/1.0
Outcome (methods) (max = 2)	2.0/2.0	2.0/1.0	2.0/2.0	2.0/2.0	2.0/2.0
Randomisation & concealment (max = 2)	1.0/1.0	0.0/0.3	0.0/0.0	0.0/1.0	0.0/0.0
Blinding (max = 3)	2.0/2.0	0.0/0.7	3.0/3.0	0.0/0.0	0.0/0.0
Number randomised (max = 2)	2.0/2.0	1.0/1.0	1.0/0.7	1.0/1.0	2.0/1.0
Analysis (max = 2)	0.0/1.3	0.0/0.3	2.0/0.7	0.0/0.3	0.0/1.0
Outcome (results) (max = 3)	3.0/3.0	1.0/1.0	3.0/3.0	3.0/3.0	3.0/3.0
Harms (max = 1)	1.0/1.0	0.0/0.0	0.0/1.0	0.0/0.0	0.0/1.0
Trial registration (max = 1)	1.0/1.0	1.0/1.0	1.0/1.0	0.0/0.0	0.0/0.0
Funding (max = 1)	1.0/1.0	0.0/0.0	0.0/0.0	0.0/0.0	0.0/0.0
Total Score (max = 25)	21.0/22.3	11.0/11.3	19.0/18.3	11.5/13.3	14.0/15.0

Values are shown as human score/mean GPT-4o score. Each item is scored on a graded scale (0–1, 0–2, or 0–3) as per the CONSORT-derived rubric, with the maximum possible score for each item shown in parentheses in the first column. Human scores represent the consensus of two expert reviewers. GPT scores are the mean of three independent runs per abstract using the final scoring prompt. Total score is the sum of the 14-item scores (maximum = 25).

### Phase 2: validation of LLM-based scoring

The validation phase evaluated the reproducibility of LLM-based scoring and its agreement with expert human ratings. The validation framework employed was grounded in established reliability and agreement methodologies widely used in instrument validation and automated scoring system evaluation. Specifically, intraclass correlation coefficients (ICC) were used to measure reproducibility^12^, while concordance correlation coefficients (CCC) and Bland–Altman analysis were applied to evaluate inter-method agreement.

Twenty additional surgical RCT abstracts were randomly sampled from surgical journals indexed in PubMed Central between 2005 and 2025, using the same eligibility criteria as in Phase 1. The validation sample size (*n* = 20 abstracts) was selected in accordance with established methodological guidance for reliability studies using intraclass correlation coefficients (ICC). Simulation and methodological studies have shown that sample sizes of 15–30 subjects with multiple ratings per subject provide stable and unbiased estimates of ICC, particularly when absolute agreement is assessed using two-way mixed-effects models^12–14^. In this study, each abstract was rated by two human experts and three independent model runs, increasing the number of observations per subject and further improving the precision of reliability estimates. The purpose of this phase was not hypothesis testing but to establish reproducibility and agreement sufficient to justify large-scale application of the automated scoring pipeline.

Abstracts and full-text PDFs were retrieved using the NCBI Entrez API via Biopython. For each article, we recorded the PMCID, title, journal, year, abstract text, and full-text introduction and methods, and stored these elements in structured JSON files. We also computed a SHA-256 hash for each abstract to provide a stable identifier and to enable later verification of content integrity.

Two expert reviewers independently scored each validation abstract using the finalised 14-item rubric. The model then scored each abstract in three independent runs using the final scoring prompt. Reproducibility of model scoring across runs was quantified using the intraclass correlation coefficient computed using a two-way mixed-effects model, single-measure, absolute-agreement definition, with ICC ≥0·75 prespecified as the threshold for acceptable reliability, following established guidance for reliability research^12^.

Agreement between mean human and mean LLM scores was assessed using the concordance correlation coefficient (CCC) and Bland–Altman analysis to estimate mean bias and limits of agreement. CCC was calculated using the bias-corrected Lin’s concordance correlation formula.

The validation phase demonstrated that the automated scoring procedure was sufficiently reproducible and concordant with expert judgement to justify its use for large-scale automated scoring and auditing. Further technical details of model calls and scoring implementation are provided in the Supplement (see Supplementary Note 7).

### Phase 3: large-scale application

During the final phase, the validated scoring and rewriting pipeline was implemented for all eligible surgical RCTs with accessible full-text articles in PubMed Central (PMC). For each included trial, the pipeline processed the original abstract and the full-text introduction and methods sections.

The model produced two rewritten abstracts, one 250 words and one 300 words. The model had to avoid fabrication, cite evidence, and follow the predefined BMRI structure, using only information from the original sources.

All outputs were logged with hash-based audit logs for traceability (see Supplementary Note 8). Reporting completeness and readability of the rewritten abstracts were compared with the original versions using the validated rubric and standard readability metrics. Results, effect sizes, and item-level changes reflect improvements achieved exclusively when full-text methods were available. This provides a consistent and interpretable estimate of large language model (LLM)-assisted reporting enhancement across open-access surgical RCTs.

### Model configuration, prompting, and safeguards

The model used was GPT-4o (OpenAI; model version gpt-4o-2024-08-06), accessed via the OpenAI API for both scoring and rewriting tasks. The model was run with deterministic configuration (temperature = 0.0), and all other parameters (including top-p, frequency penalty, and presence penalty) were left at API default values. No fallback or alternative model versions were used during the study. Model version identifiers were logged with each API call to ensure traceability. All API calls were conducted between 9 November 2025 and 10 November 2025 (UTC). No fine-tuning or parameter modification was performed.

Prompts were delivered as a structured system and user messages. The scoring prompt presented the rubric, instructed the model to return a numerical score for each of the rubric items, and required verbatim text excerpts from the abstract as evidence. The rewriting prompt provided the original abstract and, the full-text. It specified the target word count, required BMRI headings, and emphasised that the model should neither fabricate nor infer trial methods or results beyond what was provided.

Several safeguards were incorporated to mitigate hallucination and to preserve traceability. Deterministic sampling ensured that repeated calls with identical inputs yielded identical outputs. Evidence-based scoring forced the model to anchor each score to specific text in the abstract, reducing the risk of speculative scoring. Absent methodological details resulted in a score of zero for the corresponding rubric item, rather than allowing for implicit inference. During development and validation, surgical experts reviewed samples of scored and rewritten abstracts to identify systematic errors, which informed further prompt refinement. A description of the prompt iterations and hallucination-mitigation strategy is provided in the Supplement (see Supplementary Notes 3–5).

### Outcomes and scoring framework

The primary outcome was the change in reporting completeness, defined as the difference in total rubric score between the original abstract and each rewritten version (250-word and 300-word). The rubric evaluates completeness of reporting rather than correctness, accuracy, or truthfulness of the underlying trial conduct or results.

Secondary outcomes were the reproducibility of LLM scoring across repeated runs (ICC), agreement between human and model scoring (CCC and Bland–Altman bias), item-level changes in reporting completeness expressed as the change in proportion of the maximum possible score per item, and differences in readability metrics between original and rewritten abstracts.

The final scoring rubric comprised 14 items aligned with CONSORT, each scored on a graded scale (0–1, 0–2, or 0–3) based on the level of detail reported. The maximum total score was 25. Graded scoring allowed partial credit for partial reporting and distinguished between absent, minimal, and complete adherence for each item, addressing limitations of binary present/absent approaches. The same rubric was used by human experts in the development and validation sets to generate gold-standard scores. Full item definitions and scoring anchors are reproduced in the Supplement (see Supplementary Note 5).

### Statistical analysis

All statistical analyses were prespecified and implemented in Python using the scipy and statsmodels libraries.

For the validation phase, ICCs (two-way mixed-effects model, absolute agreement) quantified the reproducibility of model scoring across three independent runs per abstract. CCCs were used to assess agreement between mean human and LLM scores, and Bland–Altman plots were constructed to visualise bias and the limits.

For large-scale applications, paired comparisons of total completeness scores and readability metrics between original and rewritten abstracts were performed using paired t-tests. Effect sizes were reported using Cohen’s d, calculated as the mean paired difference divided by the standard deviation of the paired differences. Ninety-five percent confidence intervals were reported for all primary estimates.

Item-level comparisons were adjusted for multiple testing using the Benjamini–Hochberg procedure to control the false discovery rate across 14 comparisons. Confidence intervals for Cohen’s *d* were estimated using bootstrap resampling (10,000 iterations). Pearson’s correlation coefficients were used to explore relationships between completeness and readability metrics.

## Supplementary information

Supplementary information

## Data Availability

This study utilised publicly accessible abstracts of surgical randomised controlled trials (RCTs) from PubMed Central. A comprehensive list of PMCID identifiers, along with all code necessary to reproduce the dataset, scoring pipeline, and analyses, is openly available in a public GitHub repository listed below. Additional requests for derived data may be directed to the corresponding author. All scripts related to data retrieval, prompting, scoring, model auditing, and statistical analysis are publicly available in a public GitHub repository (https://github.com/Obafunsho/LLM_Surgical_RCT_Abstracts). A version-locked archival snapshot of the repository corresponding to the analyses reported in this study has been deposited in Zenodo to ensure long-term reproducibility (DOI: 10.5281/zenodo.18862160).

## Code availability

All scripts related to data retrieval, prompting, scoring, model auditing, and statistical analysis are publicly available in a public GitHub repository (https://github.com/Obafunsho/LLM_Surgical_RCT_Abstracts). A version-locked archival snapshot of the repository corresponding to the analyses reported in this study has been deposited in Zenodo to ensure long-term reproducibility (10.5281/zenodo.18862160).
