# Supplementary material for: Feasibility and impact of a large language model pipeline for surgical trial abstracts
Source: NPJ Digit Med. 2026 May 26;9:640. doi: 10.1038/s41746-026-02788-y (PMC13487255; doi:10.1038/s41746-026-02788-y)
Supplement: Supplementary file 1 [file 41746_2026_2788_MOESM1_ESM.pdf]

Feasibility and impact of a large language model pipeline for surgical trial abstracts

Authorship:

Obafunsho John Abiola[1], Dmitri Nepogodiev[1], James Glasbey[1], Omar Omar[1], Cortland Linder[1], Virginia Ledda[1], Aneel Bhangu[1*]

[1]Surgical Data Institute, University of Birmingham, Birmingham, UK

Corresponding author:

Aneel Bhangu

a.a.bhangu@bham.ac.uk

Table of Contents

SUPPLEMENT **3**
SUPPLEMENTARY NOTE 1 4
SUPPLEMENTARY NOTE 2 9
Data sources and selection *9*
Full search strategy *9*
SUPPLEMENTARY NOTE 3 12
SUPPLEMENTARY NOTE 4 14
SUPPLEMENTARY NOTE 5 16
SUPPLEMENTARY NOTE 6 18
SUPPLEMENTARY NOTE 7 24
SUPPLEMENTARY NOTE 8 25
SUPPLEMENTARY NOTE 9 26
1. Background and Rationale *26*
2. Objectives *26*
3. Study Design Overview *27*
4. Data Sources *27*
5. Phase 1 – Development Phase *28*
5.1 Sample 28
5.2 Human Scoring 28
5.3 LLM Scoring 28
5.4 Output of Phase 1 28
6. Phase 2 – Validation Phase *28*
6.1 Sample 28
6.2 Scoring 29
6.3 Statistical Validation 29
6.4 Output of Phase 2 29
7. Phase 3 – Large-Scale Application *29*
7.1 Dataset Acquisition 29
7.2 Rewriting Procedure 29
7.3 Scoring of Outputs 30
8. Outcomes *30*
8.1 Primary Outcomes 30
8.2 Secondary Outcomes 30
9. Statistical Analysis Plan *30*
9.1 Descriptive Analyses 30
9.2 Comparative Analyses 30
9.3 Reproducibility and Agreement 31
9.4 Correlation Analyses 31
9.5 Missing Data 31
10 Hallucination Mitigation and Oversight *31*
11 Reproducibility and Version Control *31*
12 Ethical Considerations *31*

Supplement

This provides detailed supplementary information to support the main manuscript entitled "Feasibility and impact of a large language model pipeline for surgical trial abstracts." It includes the TRIPOD-LLM checklist, expanded methodological details, the full rewriting and scoring prompts, the 14-item scoring rubric, the full tables from the study, and an overview of the computational pipeline and code.

Supplementary Note 1

The TRIPOD-LLM guideline extends the original TRIPOD statement to research involving large language models (LLMs). It emphasises transparency, reproducibility and human oversight across the research workflow. Table 1: Summarises how each core Tripod-LLM item in the main manuscript

Item	Domain / section	TRIPOD-LLM item	How addressed in this study	Manuscript location
1	Title & abstract	Identify use of an LLM in the title	Title explicitly states that a large language model is used to improve completeness of surgical RCT abstracts (GPT-4o, OpenAI).	Title page
2	Title & abstract	Abstract summarises model, data, evaluation, and key findings	Abstract reports data source (surgical RCTs, PubMed Central 2005–2025), LLM (GPT-4o, OpenAI), CONSORT-derived rubric, validation metrics (ICC, CCC), and main improvements in completeness and readability.	Abstract
3	Introduction	Background and rationale for LLM use	Introduction explains poor CONSORT adherence in surgery, limits of naïve LLM generation, and the rationale for a constrained, auditable LLM pipeline as a potential solution.	Introduction, paragraphs 1–2
4	Introduction	Clear objectives	Primary aim: evaluate whether an LLM can enhance reporting completeness and readability of surgical RCT abstracts. Secondary aims: develop and validate a rubric, assess reproducibility, agreement, and item-level changes.	Introduction (final paragraph); Methods, Study design
5	Data	Data source and timeframe	Surgical RCT abstracts obtained from PubMed Central, 1 January 2005 to 31 December 2025, using Entrez API and Biopython.	Methods, Study design; Phase 3 dataset
6	Data	Eligibility criteria	Inclusion: English-language RCTs of surgical or perioperative interventions with full-text PDF. Exclusion: non-randomised, non-human, reviews, protocols, observational studies.	Methods, Phase 1 and Phase 3 data sources
7	Data	Data preprocessing and representation	Abstracts, selected full-text sections, and metadata (PMCID, year, journal, title) stored as structured JSON, each record hashed with SHA-256 for integrity.	Methods, Dataset acquisition; Computational workflow
8	Model	LLM specification	The model used was GPT-4o (OpenAI; model version gpt-4o-2024-08-06), accessed via the OpenAI API. All analyses were conducted using	Methods, Model configuration

Item	Domain / section	TRIPOD-LLM item	How addressed in this study	Manuscript location
			this fixed model version. No fine-tuning or parameter modification was performed.	
9	Model	Prompt description	Two prompt types: scoring prompt with 14-item rubric, item-wise instructions, JSON schema, and evidence citation; rewriting prompt with Background–Methods–Results–Interpretation structure and non-fabrication rules.	Methods, Prompting methodology; Safeguards; Supplement (supplementary note 4)
10	Model	Safety constraints and hallucination mitigation	Prompts prohibit invention, require verbatim citations for scoring, instruct omission rather than inference when information is missing, and use full-text grounding where needed.	Methods, Safeguards; Discussion (limitations)
11	Development	Development dataset description	Five surgical RCT abstracts sampled from high-impact journals for rubric and prompt development; scored by two surgeons and GPT-4o across three runs.	Methods, Phase 1 development
12	Development	Use of development data	Human–LLM discrepancies used to refine rubric anchors, clarify ambiguous items (blinding, numbers analysed), and tighten prompt wording and JSON schema.	Methods, Phase 1 development
13	Validation	Validation dataset and purpose	Twenty additional abstracts sampled for validation; scored by surgeons and GPT-4o to estimate reproducibility and human–LLM agreement before large-scale application.	Methods, Phase 2 validation
14	Validation	Performance metrics and thresholds	Pre-specified ICC≥0·75 as acceptable reliability; used ICC (two-way mixed, absolute agreement), CCC, and Bland–Altman bias and limits of agreement.	Methods, Statistical analysis
15	Outcomes	Primary and secondary outcomes	Primary outcome: change in total rubric score between original and rewritten abstracts. Secondary: reproducibility, agreement, item-level changes, readability metrics, exploratory correlations and preferences.	Methods, Outcomes
16	Reproducibility & traceability	Versioning (model, rubric, prompts)	Model version, rubric version, and prompt version recorded and stored along with each JSON output, enabling reconstruction of any run.	Methods, Safeguards; Computational workflow

Item	Domain / section	TRIPOD-LLM item	How addressed in this study	Manuscript location
17	Reproducibility & traceability	Hashing, caching, and audit trail	SHA-256 hash computed for all abstracts and outputs; caching used to avoid repeated calls on identical inputs; all prompts and responses stored to create a complete audit trail.	Methods, Computational workflow; Safeguards
18	Human oversight	Role of human experts	Surgeons designed and refined rubric, scored development/validation sets, inspected failure cases, and participated in blinded preference assessments of original versus rewritten abstracts.	Methods, Phase 1–2; Safeguards; Discussion
19	Large-scale application	Description of large-scale dataset and pipeline	A total of 1,066 surgical RCTs were initially retrieved from PubMed Central. Of these, 651 had an accessible full-text XML record in PMC and formed the full-text analysis cohort. Articles lacking an accessible PMC full text were excluded from rewriting and subsequent scoring. 651 eligible surgical RCT abstracts processed through the pipeline: extraction, baseline scoring, rewriting at 250 and 300 words, and re-scoring for completeness and readability.	Methods, Phase 3 large-scale application; Figure 1
20	Large-scale application	Model outputs and transformations	For each abstract, GPT-4o produced two BMRI-structured rewrites. Outputs stored as JSON with scores, evidence citations, and readability metrics computed via textstat.	Methods, Phase 3; Computational workflow
21	Results	Flow of data and analyses	Flow from identification, inclusion, development/validation subsets, and large-scale application depicted conceptually in Figure 1; numbers of abstracts per phase reported in Results.	Results, Development and validation; Figure 1
22	Results	Model performance in validation	ICC 0·94 (95% CI 0·84–0·98) for reproducibility between humans; ICC 0·91 (95% CI 0·80–0·96) for reproducibility across runs; CCC 0·71 (95% CI 0·44–0·86) versus human scores; small negative bias in Bland–Altman analysis.	Results, Development and validation
23	Results	Impact of model on main outcomes	Reporting completeness improved for all 14 items; mean total score increases of 5·70 and 6·29 points for 250- and 300-word rewrites; readability improved and word count decreased.	Results, Item-level effects; Completeness; Readability

Item	Domain / section	TRIPOD-LLM item	How addressed in this study	Manuscript location
24	Results	Error modes and qualitative observations	Over-scoring occasionally observed for blinding and numbers analysed before prompt refinement; qualitative review showed some nuanced human–model differences in interpreting implicit reporting.	Results, Development; Human–model agreement
25	Discussion	Interpretation of findings	Discussion summarises that a constrained LLM can improve CONSORT completeness and readability in surgical RCT abstracts, with surgery as a multispecialty exemplar.	Discussion, paragraph 1
26	Discussion	Relation to prior evidence	Compares findings with prior work on CONSORT adherence and LLM use in abstracts, including studies where naïve LLM outputs were more readable but less compliant or fabricated details.[1–6]	Discussion, paragraph 2
27	Discussion	Limitations, risks, and generalisability	Details hallucination risk, scope limited to surgical RCTs and English-language open-access abstracts, evaluation of a single model, focus on completeness versus accuracy, automation bias, and broader ethical concerns.[8–11]	Discussion, limitations paragraph
28	Discussion	Implications for practice and policy	Outlines potential use by authors, peer reviewers, and editors; role in global surgery and equity; use in monitoring adherence to reporting standards over time.	Discussion, implications paragraph
29	Discussion	Future research directions	Proposes validation in other specialties and languages, evaluation of other models, randomised integration into editorial workflows, and extension to full manuscripts and other reporting guidelines.[7]	Discussion, future research paragraph
30	Other	Funding and competing interests	Declares that no funding was received and that authors have no competing interests.	Funding and conflicts statements
31	Other	Data and code availability	States that PMCID list and all code for retrieval, scoring, rewriting, and analysis are available in a public GitHub repository; full texts not redistributed due to copyright.	Data and code availability statement

Item	Domain / section	TRIPOD-LLM item	How addressed in this study	Manuscript location
32	Other	Reporting checklist	States that the study was designed and reported in accordance with TRIPOD-LLM and that a completed checklist is provided in the Supplement (Checklist S1).	Methods, Study design; Supplement Checklist S1

Supplementary Note 2

Data sources and selection

The development set comprised five open-access surgical randomised controlled trial (RCT) abstracts. These were manually scored by two surgical experts to refine the scoring rubric and prompts. The validation set contained 20 additional surgical RCT abstracts sampled from high-impact journals between 2005 and 2025. Eligibility criteria excluded protocols, observational studies, non-human research, case reports and meta-analyses. Full texts and abstracts were retrieved via the NCBI Entrez API, and each abstract was linked to its PubMed Central identifier.

For the large-scale application, eligible surgical RCT abstracts were identified via a structured search of PubMed (pubmed.ncbi.nlm.nih.gov) and retrieved from PubMed Central (PMC) using the NCBI Entrez API. Each abstract was stored as a JSON file containing the original abstract text, full text if available, and metadata (PMCID, title, journal, year). The downloads.py script handled retrieval, retries for failed downloads, and logging of data sources.

Full search strategy

The following search string was entered into the PubMed search interface. The Full Free Full Text filter (ffrft) was applied to restrict retrieval to articles with freely available full text in PubMed Central. The search was limited to articles published between 2005 and 2025.

```
("Surgical Procedures, Operative"[MeSH Terms]
 OR "General Surgery"[MeSH Terms]
 OR "Specialties, Surgical"[MeSH Terms]
 OR surg*[tiab] OR operativ*[tiab] OR operat*[tiab]
 OR resect*[tiab] OR laparoscop*[tiab] OR laparotom*[tiab]
 OR incision*[tiab] OR excis*[tiab] OR repair*[tiab]
 OR anastomos*[tiab] OR graft*[tiab] OR flap*[tiab]
 OR "surgical approach"[tiab])
AND
  ("Randomized Controlled Trial"[Publication Type]
   OR "Randomised Controlled Trial"[Publication Type]
   OR "Clinical Trial"[Publication Type])
NOT
  ("Review"[Publication Type]
   OR "Meta-Analysis"[Publication Type]
   OR "Systematic Review"[Publication Type]
   OR "Scoping Review"[Publication Type]
   OR "Case Reports"[Publication Type]
   OR "Letter"[Publication Type]
   OR "Editorial"[Publication Type]
   OR "Comment"[Publication Type]
   OR "News"[Publication Type]
```

```
   OR "Guideline"[Publication Type]
   OR "Practice Guideline"[Publication Type]
   OR "Consensus Development Conference"[Publication Type]
   OR "Observational Study"[Publication Type]
   OR "Cohort Studies"[MeSH Terms]
   OR "Case-Control Studies"[MeSH Terms]
   OR "Cross-Sectional Studies"[MeSH Terms]
   OR protocol[Title] OR "study protocol"[Title]
   OR pilot[Title] OR "pilot study"[Title]
   OR "long-term follow-up"[Title]
   OR "follow-up study"[Title]
   OR "qualitative research"[MeSH Terms]
   OR qualitative[tiab] OR "case series"[tiab]
   OR anaesthe*[tiab] OR anesthe*[tiab]
   OR "Endoscopy"[MeSH Terms] OR endoscop*[tiab]
   OR bronchoscop*[tiab] OR colonoscop*[tiab]
   OR cystoscop*[tiab])
AND humans[MeSH Terms]
AND
  ("JAMA Surgery"[Journal]
   OR "Endoscopy"[Journal]
   OR "International Journal of Surgery"[Journal]
   OR "British Journal of Anaesthesia"[Journal]
   OR "Anesthesiology"[Journal]
   OR "The British Journal of Surgery"[Journal]
   OR "American Journal of Transplantation"[Journal]
   OR "Hepatobiliary Surgery and Nutrition"[Journal]
   OR "Journal of Neurology, Neurosurgery, and
Psychiatry"[Journal]
   OR "Anaesthesia"[Journal]
   OR "European Journal of Vascular and Endovascular
Surgery"[Journal]
   OR "European Journal of Anaesthesiology"[Journal]
   OR "Annals of Surgery"[Journal]
   OR "Korean Journal of Anesthesiology"[Journal]
   OR "The Journal of Heart and Lung Transplantation"[Journal]
   OR "World Journal of Emergency Surgery"[Journal]
   OR "Pain"[Journal]
   OR "Arthroscopy"[Journal]
   OR "Journal of Clinical Anesthesia"[Journal]
   OR "Knee Surgery, Sports Traumatology, Arthroscopy"[Journal]
   OR "Transplantation"[Journal]
   OR "Digestive Endoscopy"[Journal]
   OR "The Journal of Bone and Joint Surgery. American
Volume"[Journal]
   OR "The Journal of Bone and Joint Surgery. British
Volume"[Journal]
   OR "BJS Open"[Journal]
   OR "Clinical Orthopaedics and Related Research"[Journal]
   OR "The Journal of Thoracic and Cardiovascular
Surgery"[Journal]
   OR "Knee Surgery & Related Research"[Journal]
   OR "Journal of NeuroInterventional Surgery"[Journal]
   OR "The American Journal of Surgical Pathology"[Journal]
   OR "Aesthetic Surgery Journal"[Journal]
   OR "Neurosurgery"[Journal]
```

```
   OR "The Annals of Thoracic Surgery"[Journal]
   OR "Liver Transplantation"[Journal]
   OR "Liver Transplantation and Surgery"[Journal]
   OR "Anesthesia and Analgesia"[Journal]
   OR "Surgery for Obesity and Related Diseases"[Journal]
   OR "Asian Journal of Surgery"[Journal]
   OR "Diseases of the Colon and Rectum"[Journal]
   OR "The Journal of Trauma and Acute Care Surgery"[Journal]
   OR "Journal of Neurosurgery"[Journal]
   OR "Journal of Vascular Surgery"[Journal]
   OR "Neurospine"[Journal]
   OR "Artificial Intelligence Surgery"[Journal]
   OR "Regional Anesthesia and Pain Medicine"[Journal]
   OR "Annals of Surgical Oncology"[Journal]
   OR "Journal of Investigative Surgery"[Journal])
AND ((ffrft[Filter]) AND (2005:2025[pdat]))
```

This search returned 1,066 records. After programmatic retrieval via the NCBI Entrez API, articles for which full-text XML could not be retrieved were logged and excluded, yielding a final dataset of 651 abstracts.

Supplementary Note 3

This section reproduces the final LLM rewriting prompt used to generate the 250- and 300-word abstracts. Earlier versions are available upon request; the final prompt incorporates lessons from iterative testing, emphasises CONSORT compliance and avoids hallucination.

```
You are an experienced researcher writing a scientific
manuscript for a surgical journal.

Below is the full manuscript including an abstract.

Your aim is to re-write the abstract so that it is compliant
with CONSORT guidelines for randomised controlled trials. Use
the attached manuscript to access information that is missing
from the current abstract.

{Full Article}
{FULL ARTICLE TEXT}

{Original Abstract}
{ ORIGINAL ABSTRACT TEXT}

The abstract should be structured with the following sections:
Background, Methods, Results, Interpretation.

Ensure your abstract includes all the following information:
- **Title:** Identify the study as a randomised controlled
trial.
- **Trial design:** State the unit of randomisation and describe
the sample size calculation.
- **Participants:** State both the participant eligibility
criteria and the study setting (i.e. where the study was
completed).
- **Intervention:** Describe both the intervention(s) and the
comparator (control).
- **Objective:** Describe the study objective.
- **Outcome:** State and define the primary outcome, including
the timeframe over which it was measured.
- **Randomisation:** State both randomisation method and
allocation concealment method.
- **Blinding (masking):** State which of the outcome assessors,
patient and clinician were blinded.
- **Number randomised:** State the total number of participants
randomised to each group and specify the period of recruitment.
- **Number analysed:** State the number analysed in each group
and whether the analysis was intention-to-treat, per-protocol or
another approach.
- **Results:** Report the main outcome's effect size and its
precision (e.g., 95 % confidence interval).
- **Harm:** Describe whether adverse events occurred, their
frequency and severity by group, and state if none were
reported.
```

```
- **Trial registration:** Provide the trial registration number
on the trial register.
- **Funding:** Report the source of funding for the trial.

Rewrite the abstract into two separate versions:
1. A 250-word version
2. A 300-word version

Separate the versions using this marker: ---

Always return integers (not strings).
Do NOT include explanations, comments, markdown or formatting.
Only use information from the existing abstract and full-text
manuscript.  Do not make any details up.
```

Supplementary Note 4

The final scoring prompt instructs GPT to evaluate each abstract against a 14-item graded rubric derived from CONSORT-for-Abstracts. The prompt requires GPT to return a strict JSON object with per-item scores and supporting evidence..

```
Here’s an abstract.  You are an expert clinical researcher
evaluating an RCT abstract using a 14-item, graded rubric.

Return **only** a strict JSON object with this schema:
{
  "rubric_version": "v3.0-2025-10-14",
  "items": {
    "<ItemName>": {"score": <int>, "evidence": "<exact
supporting phrase/sentence or Not reported>"}
    // include ALL items listed below
  },
  "total_score": <int>,
  "max_score": 25
}

Rules:
- Scores must be integers in the allowed range for each item.
- Evidence must be an exact phrase or sentence from the abstract
that supports the score; if absent, use “Not reported”.
- Do **not** include any commentary, markdown, code fences or
explanations—return the JSON only.

Rubric (14 items):
– **Title** (0–1): 0 if the title does not identify a randomised
trial; 1 if it does.
– **Trial Design** (0–2): 0 if no design; 1 if unit of
randomisation stated; 2 if unit and sample size calculation
stated.
– **Participants** (0–2): 0 if neither eligibility criteria nor
study setting given; 1 if either eligibility criteria or study
setting given; 2 if both given.
– **Intervention** (0–2): 0 if no description; 1 if intervention
described; 2 if both intervention and comparator described.
– **Objective** (0–1): 0 if objective not stated; 1 if objective
stated.
– **Method_Outcome** (0–2): 0 if no outcome description; 1 if
primary outcome stated but no timeframe; 2 if primary outcome
and timeframe stated.
– **Randomisation_Allocation** (0–2): 0 if neither randomisation
nor allocation concealment described; 1 if either randomisation
method or allocation concealment described; 2 if both described.
– **Blinding** (0–3): 0 if no blinding reported; 1 if one of
outcome assessor, patient or clinician reported; 2 if two of
these reported; 3 if all three reported or explicit mention of
triple-blinding/unblinded/open-label.
– **Number Randomised** (0–2): 0 if numbers not reported; 1 if
numbers per group reported; 2 if numbers per group and
recruitment period reported.
```

```
- **Number Analysed** (0–2): 0 if numbers analysed not reported;
1 if either numbers analysed per group or analysis set (e.g.,
intention-to-treat) reported; 2 if both reported.
- **Result_Outcome** (0–3): 0 if no results; 1 if numerical
results by group but no effect size; 2 if results and effect
size but no precision; 3 if results, effect size and precision
(e.g., 95 % CI).
- **Harms** (0–1): 0 if adverse events not reported; 1 if
reported.
- **Trial Registration** (0–1): 0 if registration not stated; 1
if registry name and number provided.
- **Funding** (0–1): 0 if funding source not reported; 1 if
reported.

Abstract to score:
<abstract text>
```

Supplementary Note 5

The scoring rubric comprises 14 items adapted from the CONSORT-for-Abstracts checklist. Each item has a maximum score and a rule for awarding points. The total score has a maximum of 25 points.

Table 2: Summarises the CONSORT-Derived scoring rubric

Item	Maximum score	Scoring rule
Title	1	0 = title does not identify randomised trial; 1 = title identifies randomised trial.
Trial Design	2	0 = no description of trial design; 1 = unit of randomisation stated; 2 = unit of randomisation and sample size calculation stated.
Participants	2	0 = no eligibility criteria or setting; 1 = either eligibility criteria or setting; 2 = both eligibility criteria and setting.
Intervention	2	0 = no description; 1 = intervention described; 2 = intervention and comparator described.
Objective	1	0 = objective not stated; 1 = objective stated.
Method Outcome	2	0 = no outcome description; 1 = primary outcome stated without timeframe; 2 = primary outcome and timeframe stated.
Randomisation & Allocation	2	0 = neither randomisation nor allocation concealment described; 1 = either randomisation or allocation concealment described; 2 = both described.
Blinding	3	0 = no blinding described; 1 = one of outcome assessor/patient/clinician blinded; 2 = two of these blinded; 3 = all

Item	Maximum score	Scoring rule
		three blinded or explicit triple-blinding/unblinded/open-label statement.
Number Randomised	2	0 = numbers not reported; 1 = numbers per group reported; 2 = numbers per group and recruitment period reported.
Number Analysed	2	0 = numbers analysed not reported; 1 = either numbers analysed or analysis set reported; 2 = both numbers analysed and analysis set reported.
Result Outcome	3	0 = no results; 1 = numerical results per group only; 2 = results and effect size; 3 = results, effect size and precision (e.g., confidence intervals).
Harms	1	0 = adverse events not reported; 1 = adverse events reported.
Trial Registration	1	0 = registration not stated; 1 = registry name and number provided.
Funding	1	0 = funding source not reported; 1 = funding source reported.

Supplementary Note 6

The scoring rubric employed in this study is an operational adaptation of the CONSORT for Abstracts checklist, rather than a novel reporting framework. To enhance transparency and reproducibility, Table 3 aligns each rubric item with its corresponding CONSORT checklist item(s). When rubric scoring consolidates or simplifies checklist components, explanatory notes clarify how the rubric translates the reporting requirements for abstract-level evaluation.

Table 3: Cross-mapping of the 14-item CONSORT scoring rubric to the CONSORT checklist

Rubric Item	Max Score	Rubric Scoring Rule	CONSORT Item(s)	CONSORT Domain	CONSORT Checklist Item Text	Notes on Correspondence
Title	1	0 = title does not identify randomised trial; 1 = title identifies randomised trial.	1a	Title and abstract	Identification as a randomised trial.	Direct 1:1 correspondence. The rubric awards 1 point when the title includes any term identifying the study as a randomised controlled trial.
Trial Design	2	0 = no design description; 1 = unit of randomisation stated; 2 = unit of randomisation and sample size calculation stated.	9, 16a	Trial design; Sample size	9: Description of trial design including type of trial, allocation ratio, and framework. 16a: How sample size was determined, including all assumptions supporting the calculation.	Rubric captures unit of randomisation (item 9) and sample size calculation (item 16a) as its two graded sub-elements. The 2025 checklist additionally requires trial framework (e.g., superiority) and allocation ratio, which are not explicitly scored.
Participants	2	0 = neither eligibility criteria nor setting; 1 = either eligibility criteria or setting; 2 = both.	11, 12a	Trial setting; Eligibility criteria	11: Settings and locations where the trial was conducted. 12a: Eligibility	Rubric directly maps eligibility criteria (item 12a) and setting/location (item 11) as its two graded sub-

Rubric Item	Max Score	Rubric Scoring Rule	CONSORT Item(s)	CONSORT Domain	CONSORT Checklist Item Text	Notes on Correspondence
					criteria for participants.	elements, consistent with the 2025 checklist.
Intervention	2	0 = no description; 1 = intervention described; 2 = intervention and comparator described.	13	Intervention and comparator	Intervention and comparator with sufficient detail to allow replication.	Rubric captures both intervention (score 1) and comparator (score 2) as graded sub-elements, consistent with item 13 which requires both to be described.
Objective	1	0 = objective not stated; 1 = objective stated.	7	Objectives	Specific objectives related to benefits and harms.	Direct 1:1 correspondence. Binary scoring reflects the checklist requirement. The rubric scores presence of any stated objective; the 2025 checklist specifies objectives should address both benefits and harms.
Method Outcome	2	0 = no outcome description; 1 = primary outcome stated without timeframe; 2 = primary outcome and timeframe stated.	14	Outcomes	Pre-specified primary and secondary outcomes, including the specific measurement variable, analysis metric, method of aggregation, and time point	Rubric captures primary outcome definition (score 1) and timeframe (score 2). Item 14 additionally requires measurement variable, analysis metric, and method of aggregation; these sub-elements are not explicitly

Rubric Item	Max Score	Rubric Scoring Rule	CONSORT Item(s)	CONSORT Domain	CONSORT Checklist Item Text	Notes on Correspondence
					for each outcome.	scored but are expected for full-credit responses.
Randomisation & Allocation	2	0 = neither described; 1 = randomisation method or allocation concealment described; 2 = both described.	17a, 18	Sequence generation; Allocation concealment	17a: Who generated the random allocation sequence and the method used. 18: Mechanism used to implement the random allocation sequence, describing steps to conceal the sequence until interventions were assigned.	Rubric directly maps sequence generation (item 17a) and allocation concealment mechanism (item 18) as its two graded sub-elements, consistent with the 2025 checklist.
Blinding	3	0 = no blinding described; 1 = one of outcome assessor/patient/clinician blinded; 2 = two blinded; 3 = all three blinded or explicit unblinded/open-label statement.	20a	Blinding	20a: Who was blinded after assignment to interventions (e.g., participants, care providers, outcome assessors, data analysts).	Rubric extends item 20a into a graded 0-3 scale, capturing each blinded party (patient, clinician, outcome assessor) as a discrete sub-element. Explicit unblinded or open-label statements are credited at maximum score, consistent with 2025 guidance that unblinded trials must state this explicitly.

Rubric Item	Max Score	Rubric Scoring Rule	CONSORT Item(s)	CONSORT Domain	CONSORT Checklist Item Text	Notes on Correspondence
Number Randomised	2	0 = numbers not reported; 1 = numbers per group reported; 2 = numbers per group and recruitment period reported.	22a, 23a	Participant flow; Recruitment	22a: Numbers of participants randomly assigned to each group. 23a: Dates defining the periods of recruitment and follow-up.	Rubric captures per-group randomisation numbers (item 22a; score 1) and recruitment period (item 23a; score 2), consistent with the 2025 checklist.
Number Analysed	2	0 = not reported; 1 = numbers analysed or analysis approach (ITT/PP) stated; 2 = both numbers analysed and analysis approach stated.	21b, 26	Statistical methods; Numbers analysed	21b: Definition of who is included in each analysis and in which group. 26: Number of participants included in the analysis and result for each group.	Rubric captures analysis set definition — ITT or per-protocol (item 21b) — and per-group numbers analysed (item 26) as its two graded sub-elements, consistent with the 2025 checklist.
Result Outcome	3	0 = no results; 1 = numerical results per group only; 2 = results and effect size; 3 = results, effect size, and precision (e.g., 95% CI).	26	Numbers analysed, outcomes and estimation	For each primary and secondary outcome: number of participants included in the analysis, result for each group, and the estimated effect size and its precision (e.g., 95% confidence interval).	Rubric decomposes item 26 into three graded levels: group results (score 1), effect size (score 2), and precision/CI (score 3), providing finer discrimination than the checklist's single item.

Rubric Item	Max Score	Rubric Scoring Rule	CONSORT Item(s)	CONSORT Domain	CONSORT Checklist Item Text	Notes on Correspondence
Harms	1	0 = adverse events not reported; 1 = adverse events reported.	27	Harms	All harms or unintended events in each group.	Rubric scores presence of any adverse event reporting (binary). Item 27 requires harms reported per group; the rubric does not explicitly require per-group attribution, representing a pragmatic simplification appropriate for abstract-level assessment.
Trial Registration	1	0 = registration not stated; 1 = registry name and number provided.	2	Trial registration	Name of trial registry, identifying number (with URL) and date of registration.	Rubric requires registry name and number for full credit. Item 2 additionally requires the registry URL and date of registration; these sub-elements are not scored, representing a pragmatic simplification for abstract-level scoring.
Funding	1	0 = funding source not reported; 1 = funding source reported.	5a	Funding and conflicts of interest	5a: Sources of funding and other support, and role of funders in the design, conduct, analysis, and	Rubric scores presence of any funding statement (binary). Item 5a additionally requires description of the funder's role; this is not scored by the

Rubric Item	Max Score	Rubric Scoring Rule	CONSORT Item(s)	CONSORT Domain	CONSORT Checklist Item Text	Notes on Correspondence
					reporting of the trial.	rubric, consistent with the limited space available in an abstract.

Supplementary Note 7

The study employed a modular pipeline to download data, rewrite abstracts, score reporting completeness and readability, and analyse results. Key components include:

1. **Data retrieval** (downloads.py): Abstracts and full texts were fetched from PubMed Central using the NCBI Entrez API. The script handles retries for failed downloads and logs provenance. Each downloaded record is stored as a JSON file containing the PMCID, abstract text, full text (if available) and metadata.

2. **Rewrite module** (rewriting.py): For each full-text abstract pair, the script invokes the OpenAI API using the fixed model version GPT-4o (OpenAI; model version gpt-4o-2024-08-06). The model was run with deterministic configuration (temperature = 0.0). All other parameters (including top-p, frequency penalty, and presence penalty) were left at API default values. The script normalises whitespace, truncates long full texts (keeping the beginning and end), sends the prompt to the model and parses the two structured abstracts separated by ---. If no delimiter is returned, the output is duplicated. Word counts are logged and diagnostics printed.

3. **Scoring module** (scoring.py): Abstracts (original or rewritten) are scored for readability (Flesch–Kincaid grade level, Flesch Reading Ease, word count) using the textstat library. A 14-item CONSORT-based rubric is implemented, and GPT is prompted with the scoring prompt to return a JSON object containing per-item scores and supporting evidence. Scores are cached locally based on a hash of the abstract and rubric version to ensure reproducibility. The model version (gpt-4o-2024-08-06) was logged with each API call to ensure traceability.

4. **Main workflow** (main.py): This script orchestrates the pipeline: downloading articles; scoring original abstracts; rewriting abstracts into 250- and 300-word versions; scoring the rewrites; and saving outputs. It records SHA-256 hashes and timestamps for each abstract, facilitating traceability. Outputs are organised into separate directories for original and rewritten abstracts and their respective scores.

Supplementary Note 8

All data files include SHA-256 hashes of the abstract text and timestamps in UTC. Rubric versions are embedded in the JSON outputs. The code uses caching to ensure that repeated scoring of the same abstract yields identical results. The model version GPT-4o (OpenAI; model version gpt-4o-2024-08-06) was fixed for all analyses and recorded with each output. Although a fallback to GPT-4o was available in the codebase to handle potential API rate limits, it was not triggered during the study. All API calls were performed between 9 November 2025 and 10 November 2025 (UTC). The total OpenAI API cost for processing the 651 abstracts (including rewriting and scoring) was $76.08 USD, corresponding to an average cost of approximately $0.12 per abstract. Costs reflect OpenAI pricing in effect at the time of analysis (9–10 November 2025) and may vary depending on future pricing structures or token usage. The repository is version-controlled, and all prompts, scores, rewritten abstracts and scripts will be made openly available upon publication. A version-locked archival snapshot of the repository has been deposited in Zenodo to ensure long-term reproducibility of the code used in this study.

Supplementary Note 9

1. Background and Rationale

Despite CONSORT guidance, scientific abstracts often omit essential methodological information. Large language models (LLMs) have the potential to improve reporting completeness; however, concerns persist regarding hallucination, reliability, and reproducibility. To date, few studies has systematically evaluated whether LLMs can autonomously rewrite journal RCT abstracts using the full text, or whether such rewrites enhance CONSORT reporting completeness as measured by a validated scoring framework.

This protocol outlines a prespecified methodological pipeline for a multistage, in-silico evaluation of LLM-based rewriting, scoring, and auditing of surgical RCT abstracts.

2. Objectives

Primary Objective

To evaluate whether a large language model improves CONSORT reporting completeness and readability in the rewriting of surgical RCT abstracts.

This protocol outlines a prespecified methodological pipeline for a multistage, in-silico evaluation of LLM-based rewriting, scoring, and auditing of surgical RCT abstracts.

Secondary Objective

1. To develop and validate a 14-item CONSORT-derived scoring rubric for evaluating reporting completeness.
2. To assess the reproducibility of LLM scoring (intraclass correlation coefficient).
3. To assess agreement between LLM and expert human scoring (concordance correlation coefficient; Bland–Altman bias).
4. To determine item-level improvements in reporting completeness following LLM-based rewriting.
5. To evaluate changes in readability, as measured by Flesch–Kincaid Grade Level, Flesch Reading Ease, and word count.

3. Study Design Overview

The study is a prospectively specified, multistage in-silico evaluation utilising a retrospective dataset of surgical RCT abstracts.

The evaluation consists of three phases:

1. Development phase – creation and refinement of rubric and prompts.
2. Validation phase – testing reproducibility and GPT–human agreement.
3. Large-scale application phase – autonomous rewriting and scoring of 651 surgical RCT abstracts.

All study phases were prespecified prior to data analysis.

4. Data Sources

- Search interface: PubMed (pubmed.ncbi.nlm.nih.gov); full texts retrieved via PubMed Central (PMC) using the NCBI Entrez API.
- Time window: 2005 – 2025.
- Study type: randomised controlled trials of surgical interventions.
- Language: English.
- Inclusion criteria:
  - The abstract describes an RCT;
  - The study involves a surgical intervention or perioperative procedure;
  - full-text PDF available;
  - abstract and metadata retrievable via the NCBI Entrez API
- Exclusion Criteria:
  - observational studies;
  - protocols;
  - non-human studies;
  - meta-analyses and systematic reviews;
  - case reports;
  - editorials or letters.
- Filter applied: Full Free Full Text (ffrft[Filter]), restricting the sample to articles with freely available full text in PMC.

For each included item, metadata such as PMCID, title, journal, year, abstract text, full-text introduction and methods, and SHA-256 hashes were stored.

5. Phase 1 – Development Phase

5.1 Sample

Five surgical RCT abstracts were randomly selected from high-impact surgical journals within the defined eligibility window.

5.2 Human Scoring

Two surgical experts independently scored each abstract using a prototype CONSORT-based rubric. Scoring discrepancies were used to refine the rubric.

5.3 LLM Scoring

GPT scored each abstract in three independent runs using a preliminary scoring prompt. Any inconsistencies informed refinement of the following:

- scoring rubric wording,
- scoring prompt structure,
- evidence-citation rules,
- hallucination-avoidance instructions.

5.4 Output of Phase 1

- A final 14-item rubric derived from CONSORT guidelines (maximum score = 25) was produced.
- A final scoring prompt was developed to return structured JSON outputs with per-item scores and verbatim evidence.
- A final rewriting prompt was established to enforce the BMRI structure and prohibit the fabrication of information.

6. Phase 2 – Validation Phase

6.1 Sample

Twenty additional surgical RCT abstracts were randomly selected from high-impact journals using the same inclusion criteria. Full texts were retrieved to enable hallucination checking.

6.2 Scoring

Each abstract was scored by:

- two human surgeon reviewers, and
- GPT, in three independent runs per abstract, using the final scoring prompt.

6.3 Statistical Validation

Pre-specified metrics:

- Reproducibility of GPT scoring:
    - Intraclass correlation coefficient (ICC), two-way mixed effects, absolute agreement.
    - Threshold for adequacy: ICC ≥ 0.75.
- GPT–Human Agreement:
    - Concordance correlation coefficient (CCC).
    - Bland–Altman mean bias and limits of agreement

6.4 Output of Phase 2

A validated scoring pipeline that meets pre-specified reproducibility thresholds was established.

7. Phase 3 – Large-Scale Application

7.1 Dataset Acquisition

Full texts were retrieved to enable hallucination checking. All eligible surgical RCT abstracts (651) were downloaded via API automation and stored as individually hashed JSON files.

7.2 Rewriting Procedure

GPT generated two rewritten versions of each abstract:

- ≈250 words (BMRI structured)
- ≈300 words (BMRI structured)

Prompts enforced:

- inclusion of all CONSORT items;

- use of original abstract plus full-text methods;
- strict prohibition on fabricating missing details;
- use of blank placeholders if data unavailable.

7.3 Scoring of Outputs

Each original and rewritten abstract underwent:

- CONSORT completeness scoring using validated LLM scoring;
- Readability scoring (Flesch–Kincaid Grade Level, Flesch Reading Ease, word count).

All outputs (rewrites + scoring) were SHA-256-hashed and timestamped.

8. Outcomes

8.1 Primary Outcomes

Change in CONSORT reporting completeness between original and rewritten abstracts.

8.2 Secondary Outcomes

- ICC for GPT reproducibility
- CCC for GPT–human agreement
- Item-level improvements (percentage of max score)
- Changes in readability metrics
- Consistency between 250- and 300-word outputs

9. Statistical Analysis Plan

All analyses were prespecified.

9.1 Descriptive Analyses

- Mean and SD for readability metrics.
- Mean completeness scores.

9.2 Comparative Analyses

- Paired t-tests for completeness and readability changes.

- Effect sizes (Cohen’s d).
- 95% confidence intervals for all estimates.

9.3 Reproducibility and Agreement

- ICC for GPT reproducibility across three runs.
- CCC for GPT–human concordance.
- Bland–Altman bias and LoA.

9.4 Correlation Analyses

- Pearson correlations between completeness and readability.

9.5 Missing Data

- Missing CONSORT items were assigned a score of zero.
- If models returned incomplete JSON outputs, repeat scoring was initiated.

10 Hallucination Mitigation and Oversight

- Full-text sections provided to GPT to reduce inference gaps.
- Prompts explicitly forbid the invention of non-existent details.
- Scoring requires verbatim-text evidence only.
- Any missing evidence triggers an automatic score = 0.
- Human surgeons spot-checked outputs across all phases.

11 Reproducibility and Version Control

- All scripts are version-controlled.
- Every abstract, rewrite, and score is hashed using SHA-256.
- Rubric version fixed.
- Model version (GPT-4o; model version gpt-4o-2024-08-06) recorded with each API call.
- Prompt versions are stored with outputs.

12 Ethical Considerations

All data were derived from publicly accessible publications; no ethical approval was required.
